# Restoration of 5-methoxytryptophan protects against atherosclerotic chondrogenesis and calcification in *ApoE*^−/−^ mice fed high fat diet

**DOI:** 10.1186/s12929-021-00771-1

**Published:** 2021-11-08

**Authors:** Guan-Lin Lee, Tsai-Lien Liao, Jing-Yiing Wu, Kenneth K. Wu, Cheng-Chin Kuo

**Affiliations:** 1 Institute of Cellular and System Medicine, National Health Research Institutes, Zhunan, Taiwan; 2 College of Life Sciences, National Tsing Hua University, Hsinchu, Taiwan; 3 Graduate Institute of Basic Medical Science, China Medical University, Taichung, Taiwan

**Keywords:** Chondrogenic differentiation, Vascular calcification, 5-Methoxytryptophan, Toll-like receptor 2

## Abstract

**Background:**

Toll-like receptor-2 (TLR2) promotes vascular smooth muscle cell (VSMC) transdifferentiation to chondrocytes and calcification in a p38 MAPK-dependent manner. Vascular 5-methoxytryptophan (5-MTP) is a newly identified factor with anti-inflammatory actions. As 5-MTP targets p38 MAPK for its actions, we postulated that 5-MTP protects against vascular chondrogenesis and calcification.

**Methods:**

High-fat diet-induced advanced atherosclerosis in mice were performed to investigate the effect of 5-MTP on atherosclerotic lesions and calcification. VSMCs were used to determine the role of 5-MTP in VSMC chondrogenic differentiation and calcification. Alizarin red S and Alcian blue staining were used to measure VSMC calcification and chondrogenic differentiation, respectively.

**Results:**

5-MTP was detected in aortic tissues of *ApoE*^−/−^ mice fed control chow. It was reduced in *ApoE*^−/−^ mice fed high-fat diet (HFD), but was restored in *ApoE*^−/−^*Tlr2*^−/−^ mice, suggesting that HFD reduces vascular 5-MTP production via TLR2. Intraperitoneal injection of 5-MTP or its analog into *ApoE*^−/−^ mice fed HFD reduced aortic atherosclerotic lesions and calcification which was accompanied by reduction of chondrogenesis and calcium deposition. Pam3CSK4 (Pam3), ligand of TLR2, induced SMC phenotypic switch to chondrocytes. Pretreatment with 5-MTP preserved SMC contractile proteins and blocked Pam3-induced chondrocyte differentiation and calcification. 5-MTP inhibited HFD-induced p38 MAPK activation in vivo and Pam3-induced p38 MAPK activation in SMCs. 5-MTP suppressed HFD-induced CREB activation in aortic tissues and Pam3-induced CREB and NF-κB activation in SMCs.

**Conclusions:**

These findings suggest that 5-MTP is a vascular arsenal against atherosclerosis and calcification by inhibiting TLR2–mediated SMC phenotypic switch to chondrocytes and the consequent calcification. 5-MTP exerts these effects by blocking p38 MAPK activation and inhibiting CREB and NF-κB transactivation activity.

**Supplementary Information:**

The online version contains supplementary material available at 10.1186/s12929-021-00771-1.

## Background

Atherosclerosis progression is accompanied by intimal calcification which has emerged as a major determinant of atheromatous plaque instability and rupture [1–4]. Atheromatous calcification poses a high risk for acute vascular events such as myocardial infarction and ischemic stroke [1]. Several reports indicate that vascular inflammation plays a major role in calcification [5, 6]. There is accumulating evidence that pro-inflammatory cytokines drive vascular smooth muscle cell (VSMC) migration and promote its phenotypic switch from contractile to osteochondrogenic phenotype which lead to vascular calcification [7–10]. Calcium is deposited in osteochondrogenic cells and released into extracellular matrix in a manner akin to bone formation [6]. We reported that toll like receptor 2 (TLR2) activation induces vascular SMC transdifferentiation into chondrocytes with calcium deposition [10]. Endogenous TLR2 activation plays a crucial role in aortic chondrogenesis and calcification in *ApoE*^*−/−*^ mice fed with high fat diet (HFD) as atheromatous calcification is abrogated in HFD-fed *ApoE*^*−/−*^*Tlr2*^*−/−*^ double-knockout mice [10]. TLR2 activation promotes atheromatous calcification via p38 MAPK signaling pathway which leads to enhanced IL-6 expression [10]. IL-6 was shown to drive VSMC migration and VSMC transdifferentiation into chondrocytes and calcium deposition [10–12].

In the present study, we investigated the influence of 5-methoxytryptophan (5-MTP) on TLR2-mediated chondrogenesis and calcification. 5-MTP is a newly discovered vascular factor which blocks macrophage activation and secretion of pro-inflammatory cytokines including IL-6 [13]. 5-MTP is synthesized from L-tryptophan through two steps of enzymatic catalysis. The initial step is catalyzed by tryptophan hydroxylase-1 (TPH-1) which converts L-tryptophan to 5-hydroxytryptophan (5-HTP) and the final step is catalyzed by hydroxyindole O-methyltransferase which converts 5-HTP to 5-MTP [14]. 5-MTP is released into extracellular milieu by a Golgi vesicular trafficking process [13]. 5-MTP was reported to inhibit IL-6, IL-1β and tumor necrosis factor α (TNFα) secretion in lipopolysaccharide (LPS)-treated murine macrophages by blocking p38 MAPK and NF-κB activation [13]. Furthermore, 5-MTP inhibits IL-1β-induced mouse vascular SMC migration and proliferation via the p38 MAPK signaling pathway [15]. In view of the inhibitory actions of 5-MTP on macrophage activation and VSMC migration via p38 MAPK, we were curious whether 5-MTP affects atheromatous calcification. We hypothesize that 5-MTP defends against atheromatous calcification by blocking VSMC migration and chondrogenic differentiation via inhibition of p38 MAPK-mediated IL-6 expression. The results show that vascular production of 5-MTP was suppressed by HFD-induced atherosclerosis in *ApoE*^*−/−*^ mice, which was restored by genetic deletion of TLR2. 5-MTP administration reduced chondrogenesis and calcification in HFD-induced atherosclerosis in *ApoE*^*−/−*^ mice and inhibited VSMC phenotypic switch to calcified chondrocytes induced by TLR2 and TLR4 activation. 5-MTP inhibits VSMC chondrocyte differentiation and calcification by blocking p38 MAPK-mediated CREB activation and IL-6 expression.

## Materials and methods

### Reagents

Pam3CSK4 (Pam3; tlrl-pms) was purchased from Invitrogen. DL-5-MTP (28052-84-8) was purchased from Sigma-Aldrich. Pure L form of 5-MTP (L-5-MTP) and 5-MTP methylester analog, L-5-MTPE were commercially synthesized by ASTATECH, USA. Antibodies for OPG (TA322994; Origene), α-SM actin (A5228; Sigma-Aldrich), SM22α (ab14106; Abcam), osterix (ab22552; Abcam), collagen II (ab34712; Abcam), SOX9 (sc-20095; Santa Cluz), aggrecan (MA3-16888; Thermo), interleukine-6 (ab6672; Abcam), pp38 (#4511; Cell Signaling Technology), p38 (sc-7194; Santa Cluz), pCREB (#9198; Cell Signaling Technology), CREB (#9197; Cell Signaling Technology), pp65 (#3033; Cell Signaling Technology), p65(#4746; Cell Signaling Technology) and β-actin (MAB1501; Millipore) were used in Western blot analysis and Immunohistochemistry. The antibody for detecting 5-MTP was customized by GeneScript, USA. Powders of Alizarin red S (ARS; A5533) and Alcian blue (A5268) were purchased from Sigma-Aldrich.

### Mouse mode of atherosclerosis and atherosclerotic calcification

Wild type, *Tlr2*^−/−^, *ApoE*^−/−^ and *ApoE*^−/−^*Tlr2*^−/−^ C57BL/6 J mice (8–10 weeks old) were subjected to an atherosclerotic intimal calcification by feeding a normal chow or high-fat diet as previously described [10]. In brief, mice were randomly fed a normal chow or a high-fat diet containing 20% fat and 0.12% cholesterol (Research Diets incorporation D12108Ci) for 12 or 20 weeks. Animal studies were followed the guidelines of experimental atherosclerosis studies described in the AHA Statement [16] and NIH Guide for Care and Use of Laboratory Animals. All experimental procedures were approved by the Institutional Animal Care and Use Committee of National Health Research Institutes, Taiwan.

Mice were treated with vehicle (saline), L-5-MTP (23.5 mg/kg), DL-5-MTP (23.5 mg/kg), or L-5-MTPE (24.7 mg/kg) by IP injection twice a week for 12 or 20 weeks. At indicated time points, the mice were killed by CO_2_ anaesthesia and blood samples were collected for cytokine analysis and 5-MTP measurement. The aortas were harvested for analysis of atherosclerotic lesion and calcification according to the guidelines of analysis of atherosclerotic lesions described in the American Heart Association Statement [16]. To more specifically measure the chondrogenic and calcific area of mouse lesion sites in the aortic arch area from the ascending aorta to the end of the arch, we made three 3-µm sections on each slide, with a total of 100 slides in a sequential manner. We picked one slide from every 10 slides and a total of 10 slides from each group were blindly subjected to ARS and Alcian blue staining. These 10 slides were quantified and the mean score of all the slides were presented.

### Cell culture and treatment

Primary VSMCs were isolated from 18.5-day postconception embryonic mouse aortas of C57BL/6 J mice (The National Laboratory Animal Center, Taipei, Taiwan) and *Tlr2*^−/−^ C57BL/6 J mice (The Jackson Laboratory) and cultured in a growth medium containing DMEM supplemented with 10% FBS, penicillin (100 U/mL), and streptomycin (100 μg/mL) as previously described [10].

VSMCs were pre-treated with or without DMSO, 5-MTP, or 5-MTPE for 30 min before treatment with endotoxin-free TE buffer, TLR2 ligands (0.1 μg/mL Pam3CSK4), or TLR4 ligand LPS from *Escherichia* coli 055: B5 (100 ng/ml; Sigma-Aldrich) in a calcifying medium consisting of DMEM supplemented with 10% FBS, penicillin (100 U/ml), streptomycin (100 μg/ml), 50 mg/L L-ascorbic acid and 2.16 g/L β-glycerophosphate (Sigma-Aldrich) for the indicated time, with treatment and medium changes every 3 days, unless specified otherwise.

### 5-MTP measurement

Plasma 5-MTP was measured using UPLC coupled with a Xevo™ triple quadrupole mass spectrometer (Waters) as previously described [17]. Vascular 5-MTP level was determined by immunohistochemistry (IHC) with anti-5-MTP antibody [13].

### Immunohistochemistry (IHC)

For detection of tissue protein expression, aortic sections were heated at 60 °C for 1 h and deparaffinized with xylene and rehydration through graded alcohols. Antigen sites were retrieved by heating the sections in an EDTA antigen retrieval buffer of pH 8 (Trilogy; Cell Marque Corporation) in an electric pressure cooker for 10 min. Sections were sequentially blocked by 3% H_2_O_2_ for 20 min and blocking buffer (5% BSA in phosphate-buffered saline with 0.1% Tween 20 (PBST)) for an additional 30 min. All antibodies described hereafter were diluted in PBS. Sections were incubated at room temperature for 2 h or 4 °C for 16 h with primary antibody and then washed in PBST. The sections were then incubated with horseradish peroxidase Labelled Polymer (Dako) for 60 min and washed 3 × times with PBST. Target protein expression levels in tissues were visualized using the DAB Chromogen system (Dako). To precisely evaluate our results, three to five sections with a constant interval of sectioning by order were used for each target. Tissues were counterstained with hematoxylin. The immunopositive areas in the aortic tissues were quantified using ImageJ software. The positive areas were quantified as the percentages of total tissue area. However, when comparing within groups of samples a few samples were considered outliers and excluded from further analyses.

### Oil red O staining

Each aorta was rinsed in 60% isopropanol and stained with 1% oil red O solution in 60% isopropanol for 10 min at room temperature. By three times of destaining with 66.6% isopropanol, the aortas were fixed and inspected under the microscope (Olympus E-330 camera with ED 50 mm f2.0 Macro lens). The oil red O-stained areas were quantified by using ImageJ software and expressed as the percentages of total tissue area.

### Chondrogenesis assay

The chondrogenesis in aortic tissues and VSMCs were determined as previously described [10]. In brief, the paraffin-embedded aortic tissues and paraformaldehyde-fixed VSMCs were stained with 1% Alcian blue (Sigma-Aldrich) dissolved in 3% acetic acid and then washed three times with 3% acetic acid. The staining images were acquired on Olympus XI71 microscope and DP70 camera. The Alcian blue areas in the aortic section were quantified as the percent positive area out of the total tissue area using ImageJ software. For quantitative analysis of Alcian blue in VSMCs, the Alcian blue was extracted with 10% SDS and absorbance at 650 nm was measured using an ELISA reader.

### Calcification assay

Aortic calcification and VSMC calcification were determined by ARS staining [10]. Aortas were perfused with saline and immersed in 10% formalin for 24 h at room temperature, and then directly stained with ARS (Sigma-Aldrich). After destaining with three times ddH_2_O, the aortas and inspected and examined under the microscope. The ARS-stained tissue was analyzed using ImageJ software. The percentage of tissue area with ARS nodules was calculated by dividing the ARS nodule area by the total area of the aorta.

To assess VSMC calcification, VSMCs were washed in PBS and fixed with 2% paraformaldehyde in PBS for 15 min. The wells were washed three times with PBS and stained with 1% ARS in ddH_2_O for 5 min at room temperature, and then washed 3 times with ddH2O. Calcified nodules in the wells were observed under a light microscope. To quantify ARS contents, ARS nodules were extracted using 10% acetic acid or cetylpyridinium chloride (CPC) and measured their absorbance at 450 or 560 nm using an ELISA reader.

### Elastin staining

Paraffin sections of the aortic arch were deparaffinized and rehydrated as described in the method of IHC. The elastin staining (Sigma; HT25A) was performed according to the manufacturer’s instructions. The plaque size for each section was determined by ImageJ software with pixel area and the result was presented as the percentage change over the HFD group.

### Migration

VSMC migration was analyzed using transwell plates with 8-μm pore size [18]. After 24 h of serum starvation (0.5% FBS in Dulbecco's modified Eagle's medium), VSMCs were treated with DMSO, Pam3 or LPS with or without 5-MTP pre-treatment for 24 h. The VSMCs were trypsinized, washed with PBS, re-suspended in medium with 0.5% FBS, and then placed in the upper chamber of 24-well transwell plates in triplicate and the bottom chambers were replaced with 0.5% FBS medium containing human PDGF-BB recombinant protein (10 ng/ml) (Peprotech) as a chemoattractant. VSMCs migrating through the filters after 4 h were quantified and normalized to the cell number with DMSO treatment.

### Specific ELISA

IL-6 levels in the culture supernatants and plasma were determined in microtiter plates (96-well) by a specific sandwich ELISA (14-7061-85 and 13-7062-85; eBioscience) as previously described [19]. OPG and RANKL in the culture supernatants were measured using an OPG- (ab100733; Abcam; or DY459; R&D) and RANKL- (ab100749; Abcam) specific detection ELISA Kit with a 96-well microtiter plate according to the manufacturer’s instructions.

### Protein extraction and Western blot analysis

Cellular proteins of VSMCs extracted by RIPA lysis buffer were resolved by 10% SDS-PAGE and transferred to polyvinylidene difluoride (PVDF) membranes as previously described [20]. They were blotted with specific antibodies according to the manufacturer’s instructions.

### Statistical analysis

All values were given as mean ± standard deviation (S.D.) or standard error of the mean (SEM). *T*-test was used to determine the statistical significance of difference between treatment and control groups, while one-way ANOVA was used to analyze multiple groups. Normality of the distribution and equal variance were tested before using *t* test or one-way ANOVA as previously described [10]. For appropriate 2-factor analysis, validating significance of data in 2 genotypes with different operations was achieved by Bonferroni post hoc test. *P* values of less than 0.05 were considered statistically significant.

## Results

### High fat diet reduces vascular and plasma 5-MTP via toll-like receptor 2

**Fig. 1:**
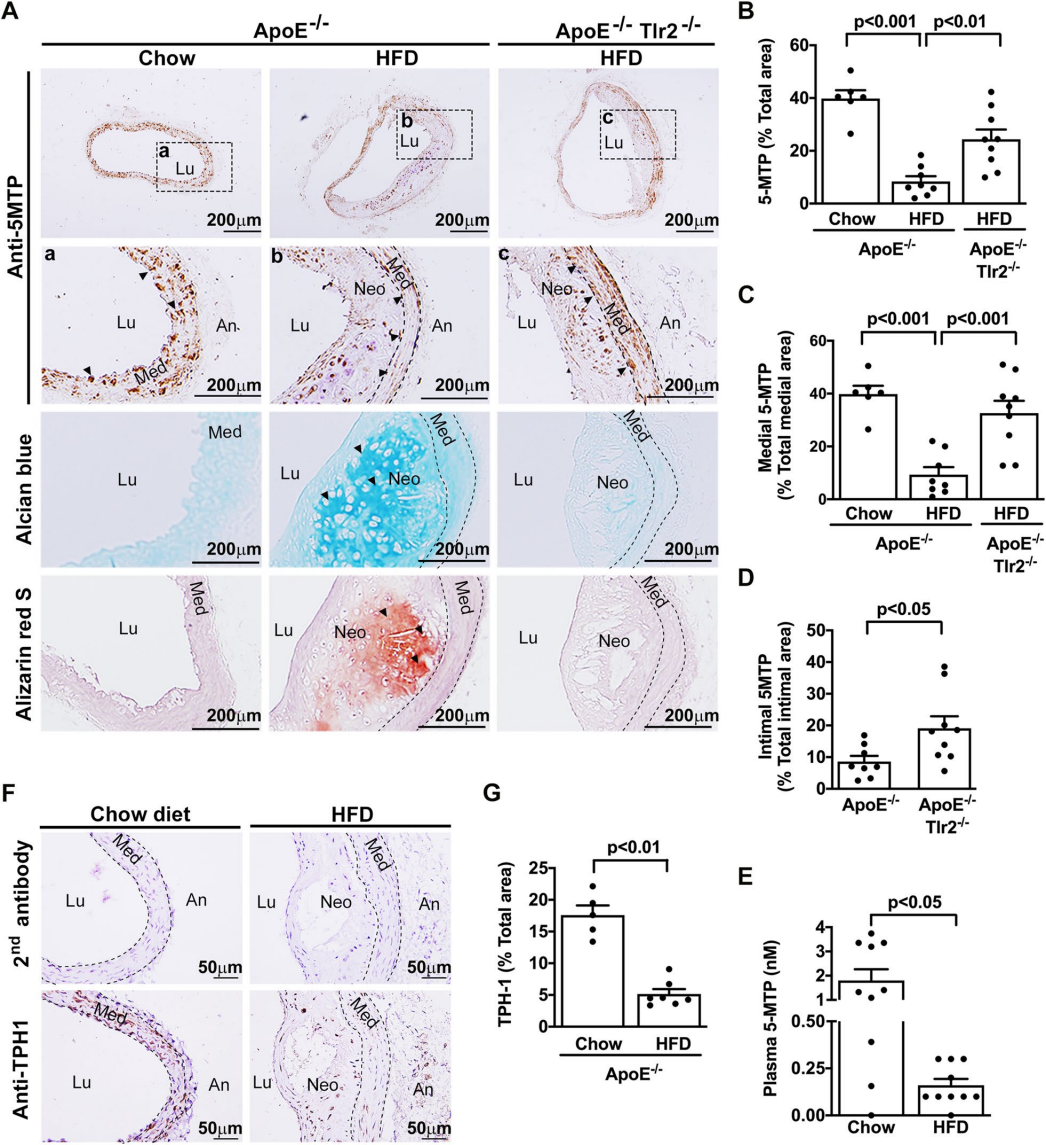
High fat diet (HFD) reduces vascular and plasma 5-MTP via TLR2. **A** Paraffin-embedded aortic tissues were prepared from chow diet–fed *ApoE*^−/−^ (n = 6), HFD-fed *ApoE*^−/−^ (n = 8), and *ApoE*^−/−^*Tlr2*^−/−^ (n = 9) mice for 20 wk and the 5-MTP contents were analyzed by immunohistochemistry as shown in the upper two panels. Insets in the second panel show the magnified views taken from the areas of aortic tissues in the first panel. Vascular chondrogenesis and calcifications in the bottom two panels were visualized with Alcian blue and ARS (ARS) staining, respectively. 5-MTP immunopositive areas in (**B**) the total artery, **C** aortic medial layer, and **D** intimal layer were measured using ImageJ software and expressed as the percent positive area out of the total tissue area. **E** Plasma 5-MTP levels in mice fed a chow diet (n = 9) or HFD (n = 20) for 20 wk were measured by quantitative LC–MS-MS. **F**, **G** TPH-1 contents in paraffin-embedded aorta tissues from mice fed a chow diet (n = 5) or HFD (n = 7) for 20 wk were analyzed by immunohistochemistry. Negative control represents staining with a 2nd antibody. Representative microphotographs are shown in (**F**) and (**G**) Immunopositive areas in the aorta tissues were quantified using ImageJ software as a percentage of the total aortic area in each section. Data in **B**–**E** and **G** represent mean ± SEM. *Lu* lumen, *Neo* neo-intima, *Med* media, *An* adventitia. *P* < 0.05, *P* < 0.01, *P* < 0.001 determined by *t*-test or a one-way ANOVA

We previously reported that vascular endothelial cells (EC) produce 5-MTP and secrete it into extracellular milieu via Golgi vesicle trafficking. Furthermore, immunohistochemistry analysis revealed positive 5-MTP staining in EC and VSMCs of mouse arteries [13]. Here, we show that *ApoE*^−/−^ mice fed regular chow retained positive 5-MTP staining in ECs and SMCs (Fig. 1A). By contrast, 5-MTP staining was significantly reduced in the atherosclerotic lesions of *ApoE*^−/−^ mice fed HFD (Fig. 1A, B), which was accompanied by inducing atherosclerotic chondrogenic differentiation and calcification (Fig. 1A, last two lower panels). Quantitative analysis confirmed significant downregulation of 5-MTP in the arterial media and intima of HFD-fed *ApoE*^−/−^ mice (Fig. 1C, D). Corresponding to 5-MTP reduction in vascular cells, plasma 5-MTP level in HFD-fed *ApoE*^−/−^ mice was significantly lower than that in *ApoE*^−/−^ mice fed control chow (Fig. 1E). As TLR2 plays a pathological role in trigging atherosclerosis and vascular calcification [10], we thus determined whether TLR2 influences 5-MTP production, we analyzed vascular 5-MTP by IHC in *ApoE*^*−/−*^*Tlr2*^−/−^ double knockout mice. 5-MTP staining in *ApoE*^*−/−*^*Tlr2*^−/−^ mice fed HFD was restored which was significantly higher than that in *ApoE*^*−/−*^ mice fed HFD (Fig. 1A–D).

5-MTP biosynthesis from L-tryptophan is catalyzed by TPH-1 [14]. We observed that TPH-1 expression in EC is suppressed by LPS and cytokines [13, 21]. We were, therefore, curious whether reduced 5-MTP synthesis in HFD-fed *ApoE*^*−/−*^ mice might be due to TPH-1 suppression. We analyzed TPH-1 expression in aortic tissues by IHC. TPH-1 expression was significantly reduced in endothelium and media of *ApoE*^*−/−*^ mice fed HFD (Fig. 1F, G). Taken together, these results reveal reduced vascular cell 5-MTP production in vascular cells of HFD-fed-*ApoE*^*−/−*^ mice via TLR2. The results suggest that 5-MTP reduction may be attributed to suppression of TPH-1 expression.

### Chondrogenesis and calcification in HFD-induced neointima are suppressed in *ApoE*^−/−^*Tlr2*^−/−^ mice

Consistent with a previous report [10], abundant chondrogenesis stained with Alcian blue was detected in the neointima of *ApoE*^−/−^ mice fed HFD which was abrogated by TLR2 deletion (Fig. 1A, lower panels). Correlated with chondrogenesis, ARS-positive calcification was detected in the region of neointima of HFD-fed *ApoE*^−/−^ mice which became undetectable in double knockout mice (Fig. 1A). Notably, HFD-mediated atherosclerotic chondrogenesis and calcification and downregulation of 5-MTP in the atherosclerotic lesion of *ApoE*^−/−^ mice was concurrently prevented in *ApoE*^−/−^*Tlr2*^−/−^ mice (Fig. 1A). These results suggest that HFD activates TLR2 to reduce vascular 5-MTP expression and the loss of 5-MTP production may subsequently resulted in atherosclerotic chondrogenesis and calcification.

### 5-MTP administration reduces chondrogenesis and calcification

**Fig. 2:**
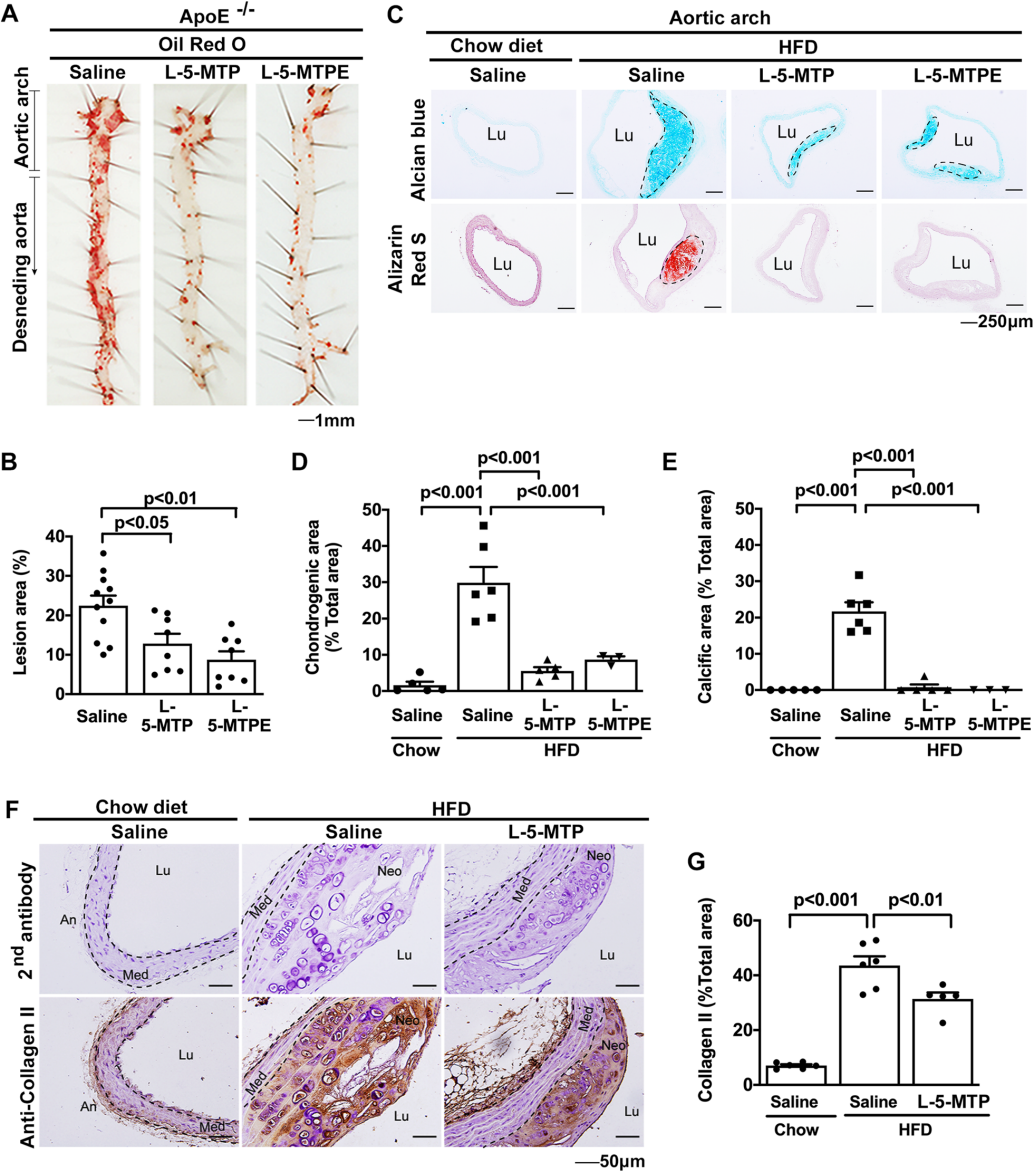
5-MTP administration reduces atherosclerotic chondrogenesis and calcification. **A**, **B** *ApoE*^*−/−*^ mice fed an HFD were received intraperitoneal injection of saline (n = 9), 5-MTP (23.5 mg/kg, n = 8), or 5-MTPE (24.7 mg/kg, n = 8) twice weekly for 12 weeks. **A** Aortic atherosclerotic lesions were evaluated by en face staining of the entire aorta with Oil Red O. **B** Oil Red O positive areas were quantified using ImageJ software as a percentage of total aortic area in each section. **C** Vascular chondrogenesis (upper panel) and vascular calcification (lower panel) in aortic arch tissues from *ApoE*^*−/−*^ mice fed chow diet with intraperitoneal injection of saline or HFD with intraperitoneal injection of saline (n = 5), 5-MTP (23.5 mg/kg, n = 5), or 5-MTPE (24.7 mg/kg, n = 3) twice weekly for 20 weeks were analyzed by Alcian blue staining and ARS, respectively. **D** Cell chondrogenic differentiation and **E** vascular calcification were quantified using ImageJ software. The percentage of tissue area with chondrogenesis or calcification was calculated by dividing the chondrogenic or calcified area by the total area of the aortic area, respectively. **F** Collagen II contents in aorta tissues from *ApoE*^*−/−*^ mice fed chow diet with saline (n = 6) or HFD with saline (n = 6) or L-5-MTP (n = 6) twice weekly for 20 wk were analyzed by immunohistochemistry. Negative control represents staining with a 2nd antibody. **G** Immunopositive areas in the aorta tissues were quantified as a percentage of the total aortic area in each section. Data in **B**, **D**–**E**, and **G** represent mean ± SEM. *Lu* lumen, *Neo* neo-intima, *Med* media, *An* adventitia. *P* < 0.05, *P* < 0.01, *P* < 0.001 determined by a one-way ANOVA

In view of the reduction of 5-MTP in HFD-induced atherosclerosis, we determine whether 5-MTP supplement protects against atherosclerotic growth and calcification. The effect of intraperitoneally administration of saline, 5-MTP, or its methylester analog, L-5-MTPE (Additional file 1: Fig. S1A) on aortic atherosclerotic lesions and calcification were evaluated by en face staining of the entire aorta with Oil Red O and ARS, respectively. L-5-MTP and L-5-MTPE reduced Oil Red O staining and atherosclerotic lesions to a comparable extent when compared to saline control (Fig. 2A, B and Additional file 1: Fig. S1B-S1C). DL-5-MTP attenuated ARS staining of the aortic arch region (Additional file 1: Fig. S1D and S1E). We next analyzed chondrogenesis and calcification by staining aortic cross-sections with Alcian Blue and ARS stain, respectively. When compared to baseline control (chow-fed *ApoE*^*−/−*^ mice), prominent Alcian Blue staining was detected in the aortic sections of *ApoE*^*−/−*^ mice fed HFD for 20 weeks which is accompanied by superimposed ARS staining (Fig. 2C–E). L-5-MTP and L-5-MTPE administration comparably reduced Alician Blue staining and abrogated ARS staining (Fig. 2C–E). As collagen II expression is a hallmark of chondrogenesis, we analyzed collagen II in aortic sections by immunohistochemistry (IHC). Compared to the collagen II staining in controls, HFD enhanced collagen II staining which was attenuated by L-5-MTP treatment (Fig. 2F, G). L-5-MTPE suppressed collagen II to an extent comparable to 5-MTP (Additional file 1: Fig. S2). Taken together, these results suggest that supplement of 5-MTP and its methylester analog L-5-MTPE to *ApoE*^*−/−*^ mice restores protection against HFD-induced aortic chondrogenesis and calcification.

**Fig. 3:**
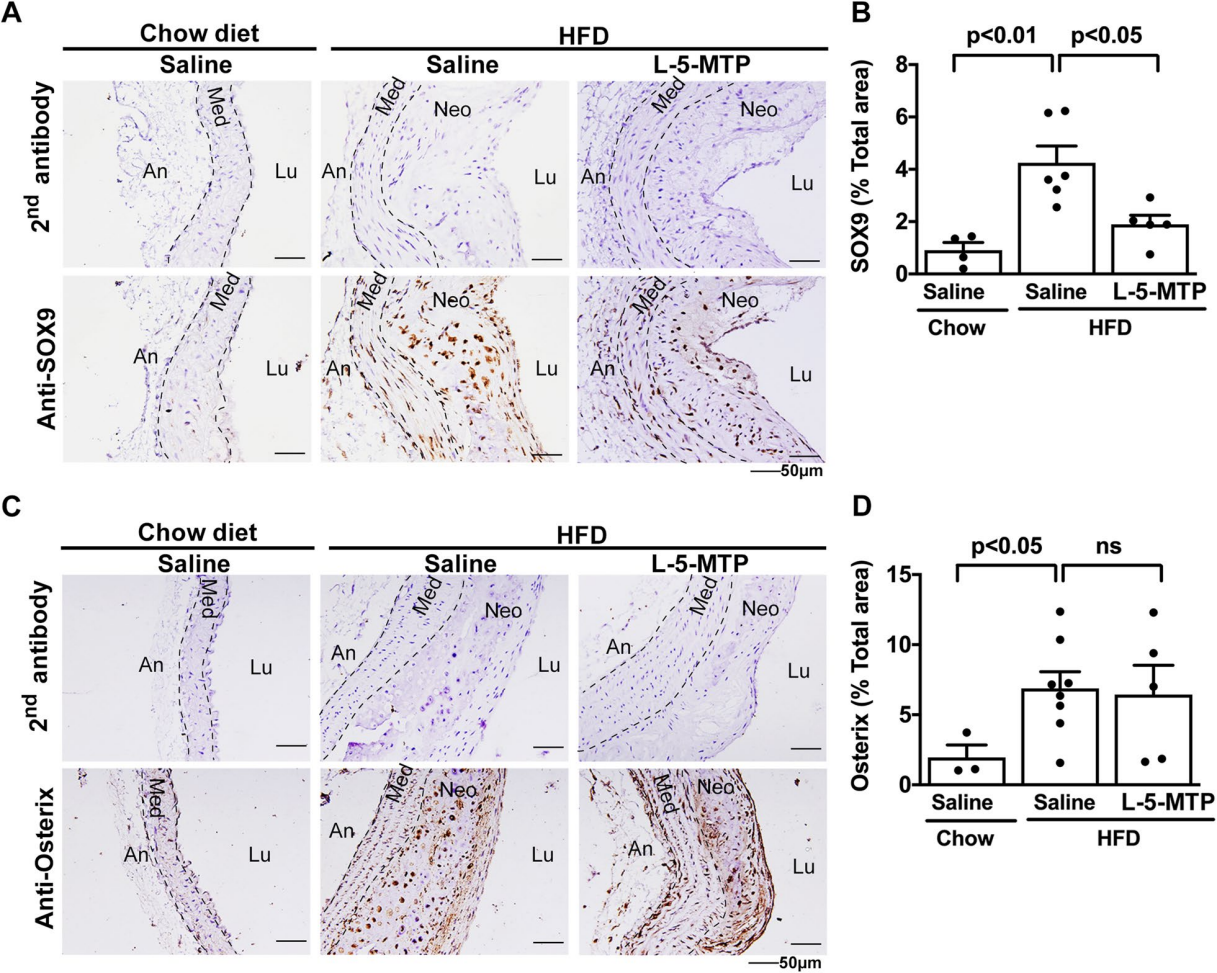
5-MTP decreases HFD-induced SOX9 expression in aortic atherosclerotic lesions. SOX9 and osterix contents in aorta tissues from *ApoE*^*−/−*^ mice fed chow diet with saline (n = 3) or HFD with saline (n = 6) or L-5-MTP (n = 5) twice weekly for 20 wk were analyzed by immunohistochemistry. Negative control represents staining with a 2nd antibody. Representative microphotographs of the immunohistochemical staining for **A** SOX9 and **C** osterix are shown. Immunopositive areas of **B** SOX9 and **D** osterix in aorta tissues were quantified as a percentage of the total aortic area in each section. Data represent mean ± SEM. *Lu* lumen, *Neo* neo-intima, *Med* media, *An* adventitia. *P* < 0.05, *P* < 0.01 determined by a one-way ANOVA

SOX9 is one of major genes driving chondrogenesis. We determine whether 5-MTP suppresses chondrogenesis through control of SOX9 expression. We analyzed SOX9 proteins in aortic tissues of HFD-fed *ApoE*^*−/−*^ mice treated with and without 5-MTP. Abundant SOX-9 proteins were detected in aortic neointima of HFD-fed *ApoE*^*−/−*^ mice treated with saline (Fig. 3A, B). SOX-9 levels were significantly lower in mice treated with 5-MTP (Fig. 3A, B) or 5-MTPE (Additional file 1: Fig. S3A and S3B). By contrast, osterix, a driver of osteogenesis, whose expression was enhanced in HFD-fed *ApoE*^*−/−*^ mice, was not affected by 5-MTP (Fig. 3C, D) or 5-MTPE (Additional file 1: Fig. S3C and S3D). These results suggest that 5-MTP blocks SOX-9 but not osterix expression, thereby decreasing chondrogenesis in the aortic atherosclerotic lesions of HFD-fed *ApoE*^*−/−*^ mice.

### 5-MTP blocks vascular SMC chondrogenic transdifferentiation and calcification induced by TLR2 activation

**Fig. 4:**
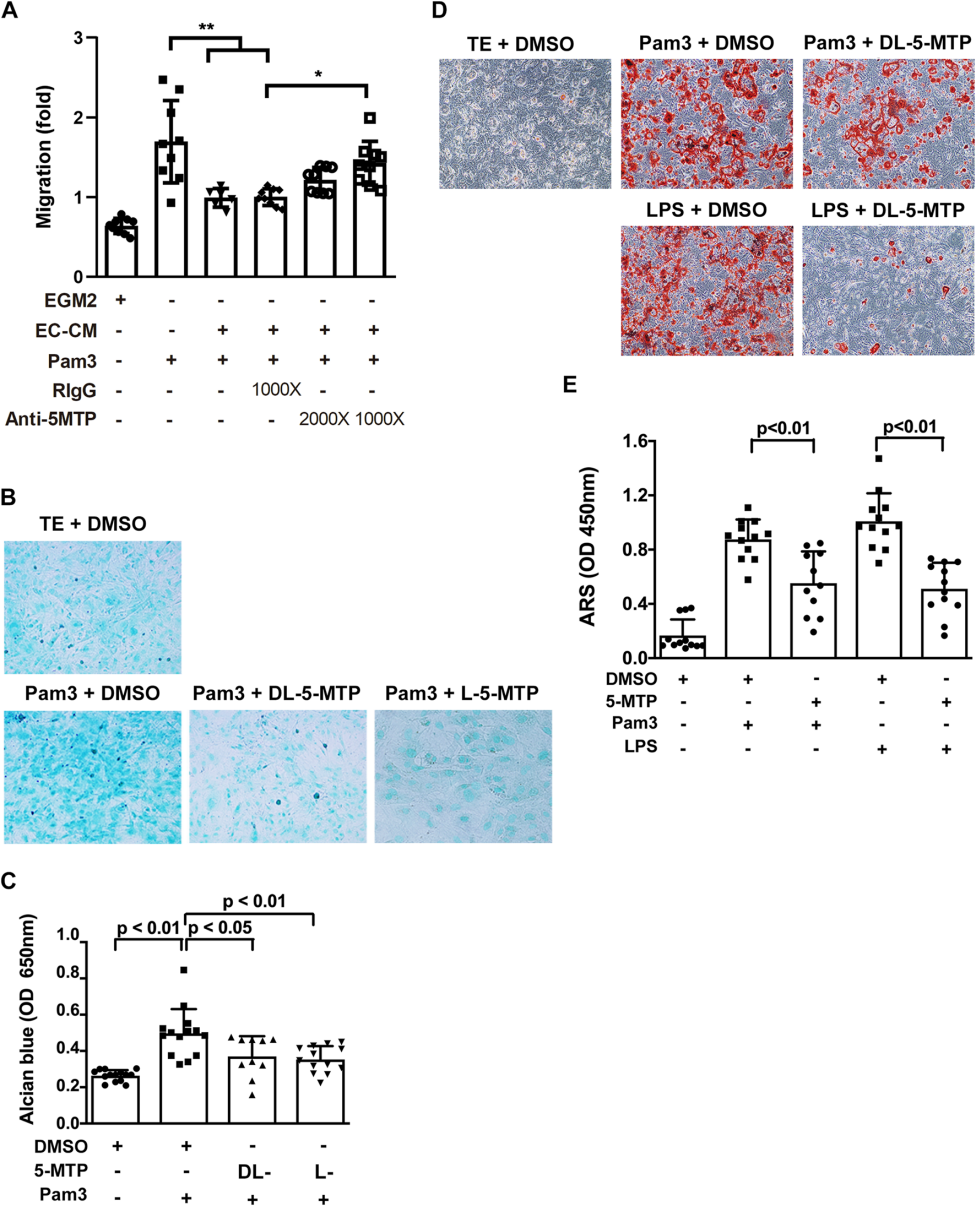
5-MTP blocks VSMC migration, chondrogenic differentiation and calcification induced by TLR2 or TLR4 agonists. **A** Quiesced VSMCs were incubated in control medium (EGM2) or conditioned medium from human aortic ECs (EC-CM) with or without Pam3CSK4 (Pam3) in the presence or absence of control IgG or anti-5-MTP antibodies for 24 h. Migration assays were then performed with PDGF-BB as a chemoattractant. VSMCs were treated with TE buffer, Pam3, or LPS in a calcifying medium with DMSO, DL-5-MTP, or L-5-MTP (100 μM) for (**B**, **C**) 2 wk or (**D**, **E**) 4 wk, with medium changes every 3 days. **B** VSMC chondrogenesis was determined by Alcian blue staining and **C** quantified by measuring Alcian blue absorbance at 650 nm. **D** VSMC calcification was determined by ARS staining and **E** quantification of calcium deposition was determined by measuring ARS nodule absorbance at 450 nm. Data represent mean ± SD of at least 3 independent experiments. *P < 0.05, **P < 0.01 determined by *t*-test or a one-way ANOVA

**Fig. 5:**
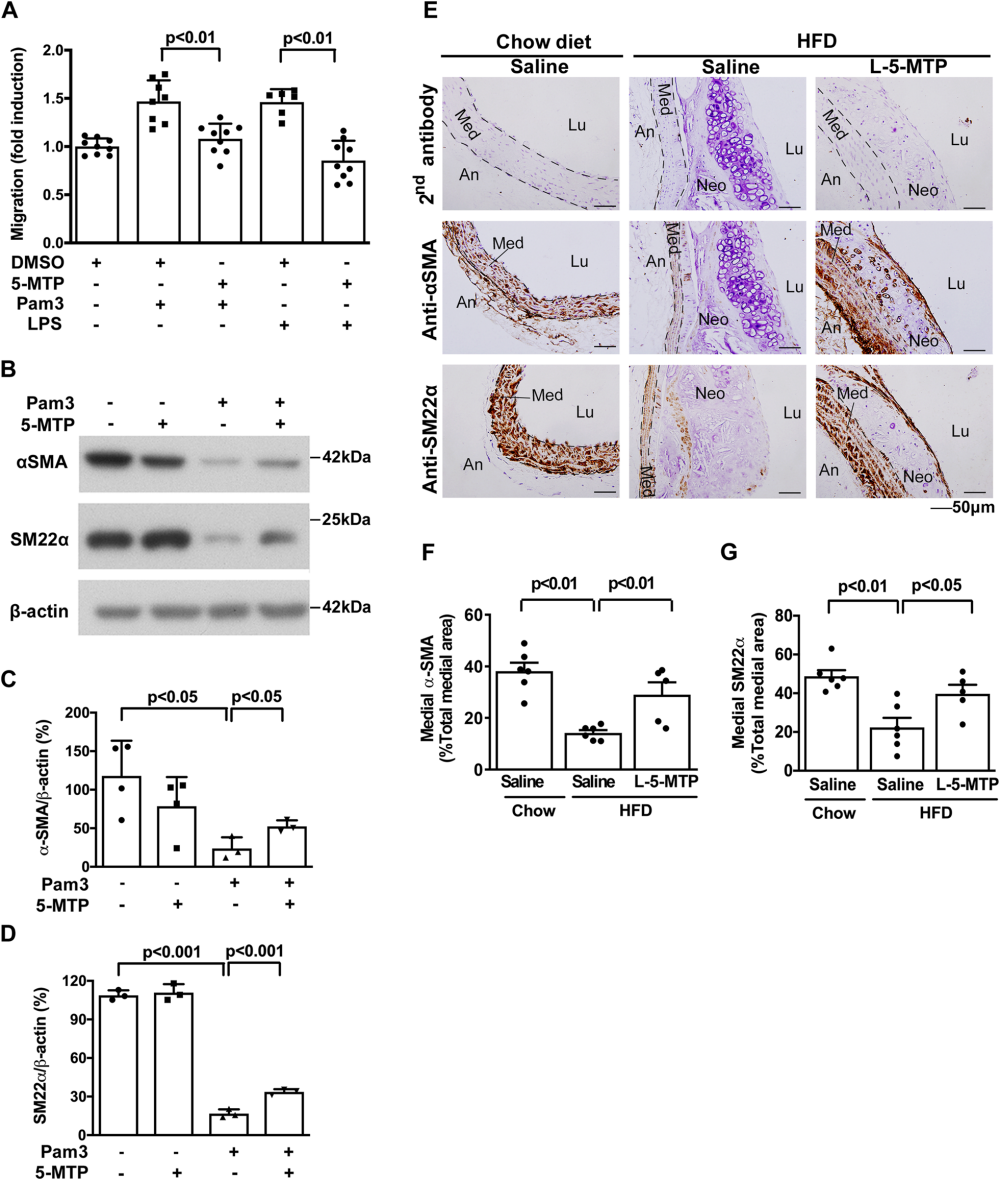
5-MTP preserves the contractile phenotype of VSMCs. After pretreating VSMCs with 5-MTP (100 μM) for 30 min, cells were stimulated with Pam3 or LPS for 24 h. **A** Migration assays were then performed with PDGF-BB as a chemoattractant. **B** Cell lysates were immunoblotted with antibodies for α-SMA (α-smooth muscle actin), SM22 α (smooth muscle protein 22 α), or β-actin. Densitometric analysis of α-SMA and SM22 α immunoblots normalized to β-actin presented in (**C**) and (**D**). Data in **A** and **C**, **D** represent mean ± SD of at least 3 independent experiments. α-SMA and SM22α contents in aorta tissues from *ApoE*^*−/−*^ mice fed chow diet with saline (n = 6) or HFD with saline (n = 6) or L-5-MTP (n = 5) twice weekly for 20 wk were analyzed by immunohistochemistry. Negative control represents staining with a 2nd antibody. Representative microphotographs are shown in (**E**). Lu, lumen; Neo, neo-intima; Med media; An, adventitia. Immunopositive areas of (**F**) α-SMA and (**G**) SM22α in the aorta tissues were quantified as a percentage of the total aortic area in each section. Data in **F**, **G** represent mean ± SEM. *P* < 0.05, *P* < 0.01, *P* < 0.001 determined by *t*-test or a one-way ANOVA

The pathological behavior and functional phenotype of VSMC are key events in the development and progression of atherosclerotic lesions. Given that endothelial cells are considered to play a key role in controlling vascular homeostasis and 5-MTP is a novel class of endothelium-derived vasoprotective molecules [13], we thus determine whether endothelial cells release 5-MTP into the conditioned medium (CM) and suppress VSMC pathophysiological function, such as VSMC migration. The results revealed that CM from human aortic endothelial cells significantly suppressed the VSMC migration induced by TLR2 agonist Pam3CSK4 (Pam3). However, this VSMC migration suppression was abrogated by anti-5-MTP antibodies but not control IgG (Fig. 4A), suggesting that endothelial cells release 5-MTP into the CM which may account for VSMC migration suppressing actions. Notably, vascular SMCs are reported to be the major cell source of chondrosteogenic transdifferentiation in atherosclerotic lesions [10]. As chondrogenesis in neointima depends on TLR2, we evaluated the effect of Pam3 on SMC chondrogenic differentiation and its influence by 5-MTP. Vascular SMCs were treated with 5-MTP followed by Pam3. Pam3-induced Alcian Blue staining was abrogated by racemic 5-MTP (DL-5-MTP) and pure L-5-MTP to a similar extent (Fig. 4B, C). VSMC calcification analyzed by ARS staining was enhanced by Pam3 and lipopolysaccharide (LPS, an activator of TLR4), and this enhancement was abrogated by DL-5-MTP (Fig. 4D and E). Pam3-induced vascular SMC transdifferentiation was accompanied by enhanced migration (Fig. 5A) and reduced expression of specific contractile proteins such as α-SMA (α-smooth muscle actin) and SM22 α (smooth muscle protein 22 α) (Fig. 5B). 5-MTP treatment slightly reduced the migration of VSMC (Additional file 1: Fig. S4A). It significantly inhibited VSMC migration induced by Pam3 and LPS in a dose-dependent manner (Fig. 5A). Furthermore, pretreatment with 5-MTP preserved partially the contractile proteins (Fig. 5B–D). LPS-induced reduction of α-SMA and SM22α was similarly blocked by L-5-MTP (Additional file 1: Fig. S4B-S4D). In addition, 5-MTP was capable of suppressing classical atherosclerotic stimuli oxLDL-induced VSMC migration (Additional file 1: Fig. S4E). To explore the in vivo relevance, we analyzed α-SMA and SM22α in aortic tissues of *ApoE*^*−/−*^ mice fed HFD vs. control chow by IHC. α-SMA and SM22α staining was diminished in HFD-fed *ApoE*^*−/−*^ mice, which was averted by 5-MTP pretreatment (Fig. 5E–G). The synthetic analog of 5-MTP, 5-MTPE, preserved the contractile phenotype when administered at an identical dosing and scheduling as 5-MTP (Additional file 1: Fig. S5). These results suggest that 5-MTP suppresses atherosclerotic calcification by preventing vascular SMC phenotypic switch and chondrogenic differentiation.

### 5-MTP suppresses IL-6 production but not osteoprotein (OPG) expression

**Fig. 6:**
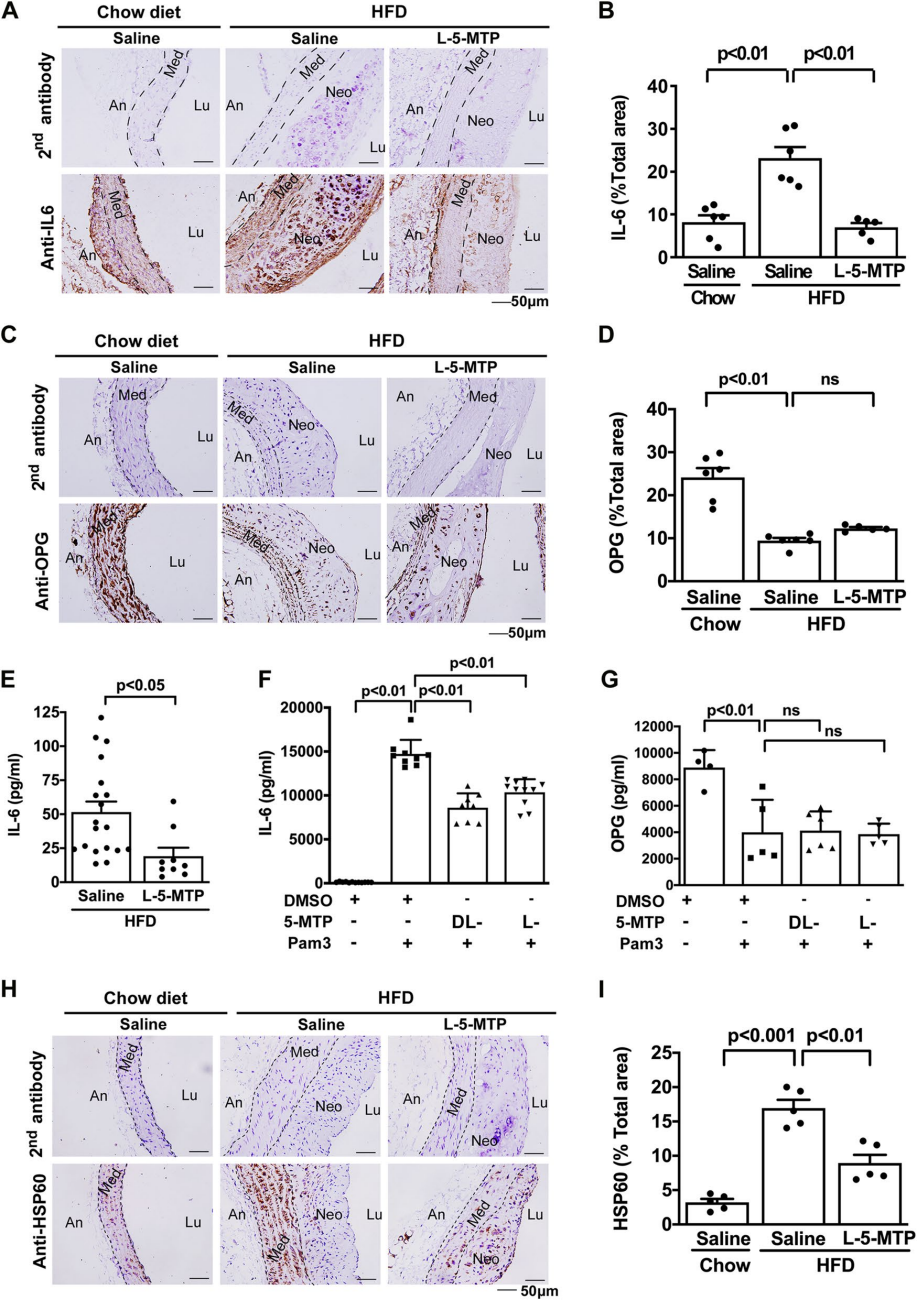
5-MTP suppresses HFD-induced IL-6 and HSP60 in the atherosclerotic lesions of *ApoE*^*−/−*^ mice. Chow diet-fed or HFD-fed *ApoE*^*−/−*^ mice were treated with saline or L-5-MTP twice weekly for 20 wk. (**A**, **B**) IL-6 and (**C**, **D**) OPG in paraffin-embedded aortic tissues were analyzed by immunohistochemistry. Negative control represents staining with a 2nd antibody. Representative microphotographs of the immunohistochemical staining for (**A**) IL-6 and (**C**) OPG are shown. Immunopositive areas of (**B**) IL-6 and (**D**) OPG in aorta tissues were quantified as a percentage of the total aortic area in each section. Data represent mean ± SEM (Chow + saline group: n = 6, HFD + saline group: n = 6 and HFD + 5-MTP group: n = 5). **E** Plasma IL-6 levels in HFD-fed *ApoE*^*−/−*^ mice with injection of saline (n = 19) or L-5-MTP (n = 9) twice weekly for 20 wk for were measured by ELISA. Data represent mean ± SEM. After pretreating VSMCs with DMSO, DL-5-MTP, or L-5-MTP (100 μM) for 30 min, VSMCs were treated with vehicle or Pam3CSK4 (Pam3) in calcifying medium for 24 h. **F** IL6 and **G** OPG level in culture supernatants was measured by ELISA. Data represent mean ± SD of at least 3 independent experiments. HSP60 contents in aorta tissues from *ApoE*^*−/−*^ mice fed chow diet with saline (n = 5) or HFD with saline (n = 5) or L-5-MTP (n = 5) for 20 wk were analyzed by immunohistochemistry. Representative microphotographs of the immunohistochemical staining (**H**) and quantification of immunopositive positive areas (**I**) are shown. Data represent mean ± SEM. *Lu* lumen, *Neo* neo-intima, *Med* media, *An* adventitia. *P* < 0.05, *P* < 0.01 determined by a one-way ANOVA

We reported previously that TLR2-induced vascular SMC chondrogenic transdifferentiation was regulated by two key factors, IL-6 and OPG [10]. We hypothesized that 5-MTP inhibits chondrogenesis by suppressing IL-6 as 5-MTP has been shown to block LPS-induced IL-6 production in murine macrophages [13]. IL-6 and OPG levels in aortic tissues of chow diet- and HFD-fed *ApoE*^*−/−*^ mice treated with and without 5-MTP were analyzed by IHC staining. HFD increased IL-6 primarily in neointima and decreased OPG in media (Fig. 6A–C). 5-MTP treatment reduced IL-6 (Fig. 6B) but had no significant effect on OPG (Fig. 6D). 5-MTP analog 5-MTPE exerted a similar effect as 5-MTP (Additional file 1: Fig. S6). In addition, L-5-MTP decreased HFD-induced plasma IL-6 level as compared with saline treatment (Fig. 6E).

We next evaluated the effect of 5-MTP on Pam3-induced release of IL-6 and OPG by VSMCs. Pam3-induced IL-6 elevation in VSMCs was significantly blocked by DL-5-MTP and L-5-MTP, whereas Pam3-induced OPG suppression was not reversed by 5-MTP (Fig. 6F, G). 5-MTP exerted a similar effect on LPS-induced IL-6 secretion. DL-5-MTP and L-5-MTP reduced LPS-induced release of IL-6 but did not affect the secretion of OPG into the medium (Additional file 1: Fig. S7). Heat shock protein 60 (HSP 60) was reported to be an endogenous ligand of TLR2 and induce inflammatory response via TLR2 [22]. We determined whether 5-MTP reduces TLR-2 mediated inflammation by blocking HSP 60. 5-MTP significantly reduced HSP 60 in aortic tissues of HFD-fed *ApoE*^*−/−*^ mice (Fig. 6H, I) and suppressed HSP 60-induced VSMC migration (Additional file 1: Fig. S8). These results suggest that 5-MTP controls TLR2-induced chondrogenesis and calcification by suppressing VSMC production of IL-6 and HSP 60 expression.

### 5-MTP suppresses p38 MAPK and CREB activation

**Fig. 7:**
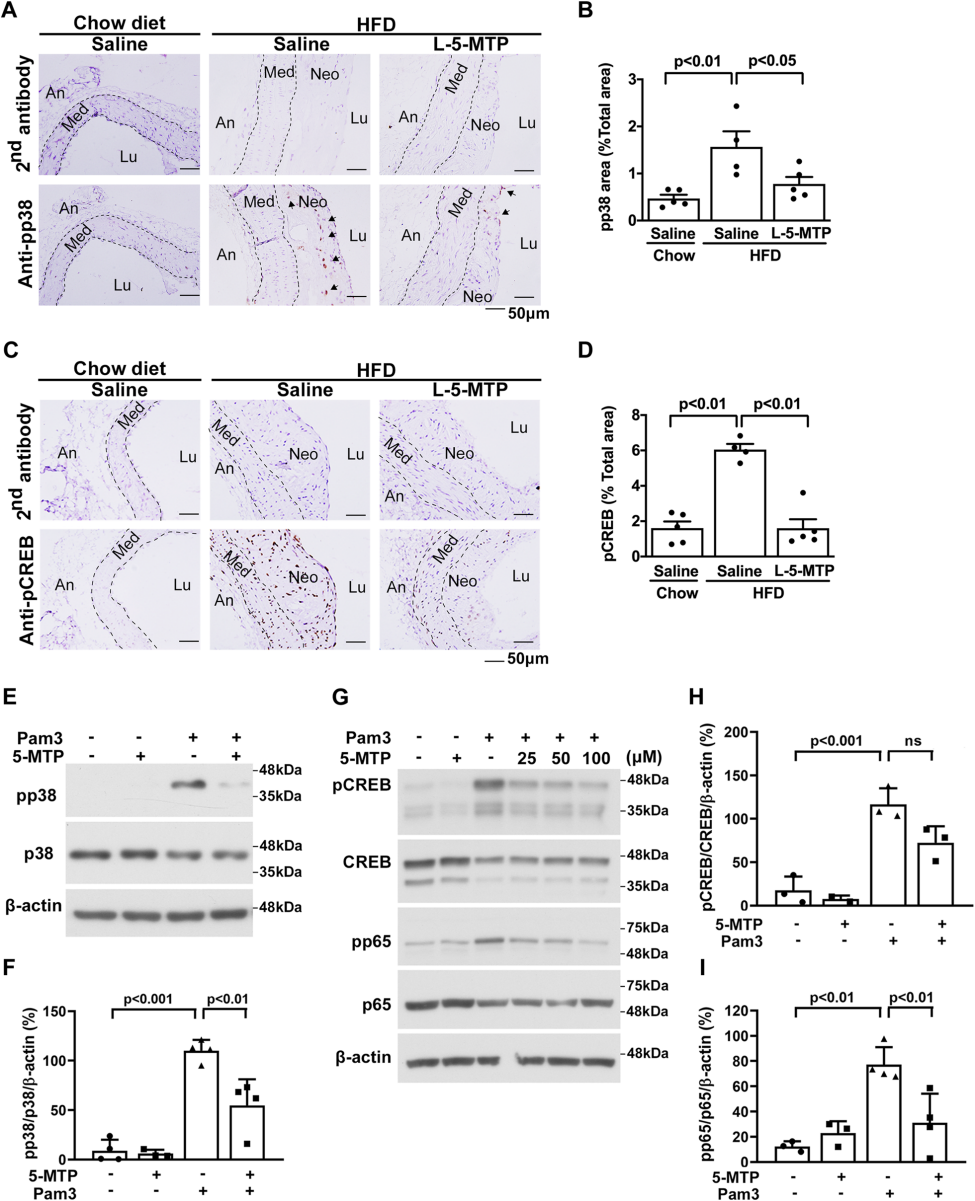
5-MTP suppresses the activation of p38 and CREB. **A**, **B** pp38 and (C-D) pCREB contents in aorta tissues from *ApoE*^*−/−*^ mice fed chow diet with saline (n = 5) or HFD with saline (n = 4) or L-5-MTP (n = 5) twice weekly for 20 wk were analyzed by immunohistochemistry. Negative control represents staining with a 2nd antibody. Representative microphotographs of (**A**) pp38 and (**C**) pCREB are shown. Immunopositive areas of (**B**) pp38 and (**D**) pCREB in the aorta tissues were quantified as a percentage of the total aortic area in each section. Data represent mean ± SEM. **E**–**I**. After pretreating VSMCs with different concentrations of L-5-MTP (**E**, **F**, 100 μM and **G**–**I**, 25 ~ 100 μM) for 30 min, cells were stimulated with Pam3CSK4 for 30 min. Cell lysates were immunoblotted with antibodies for pp38, p38, pCREB, CREB, pp65, p65, or β-actin. Densitometric analysis of immunoblots normalized to β-actin presented in (**F**) and (**H**, **I**). Data represent mean ± SD of at least 2 independent experiments. *P* < 0.05, *P* < 0.01, *P* < 0.001 determined by *t*-test or a one-way ANOVA

TLR2-mediated arterial calcification is signaled via p38 MAPK-mediated IL6 production [10]. Since 5-MTP is known to control vascular cell and macrophage activation through inhibiting p38 MAPK [13, 15, 21], we determined whether 5-MTP blocks p38 MAPK activation in HFD-fed *ApoE*^*−/−*^ mice. 5-MTP pretreatment abrogated p38 MAPK activation in neointima as shown by reducing pp38 staining (Fig. 7A, B). As p38 MAPK mediates transcription of *Il-6* via several transcriptional activators notably CREB, we evaluated the effect of 5-MTP on CREB phosphorylation in aortic tissues of HFD-fed *ApoE*^*−/−*^ mice. 5-MTP abrogated CREB phosphorylation (Fig. 7C, D). We next evaluated the effect of 5-MTP on pp38 and pCREB in cultured vascular SMCs treated with 5-MTP followed by Pam3. 5-MTP suppressed Pam3-induced pp38 MAPK (Fig. 7E, F and Additional file 1: Fig. S9), and blocked Pam3-induced CREB phosphorylation (Fig. 7G, H). NF-κB is a master regulator of transcription of pro-inflammatory genes. It was previously reported that 5-MTP inhibits phosphorylation of p65 subunit of NF-κB [13]. We evaluated the effect of 5-MTP on Pam3-induced p65 phosphorylation (pp65). Pam3-induced vascular SMC pp65 was inhibited by 5-MTP in a concentration-dependent manner (Fig. 7G, I). These results indicate that 5-MTP inhibits TLR2 signaling in VSMCs by blocking activation of p38 MAPK-driven CREB and NF-κB transactivation activity.

## Discussion

5-MTP is a newly identified factor with anti-inflammatory properties. It was reported to be produced by cultured vascular EC and SMC [13]. Immunohistochemical analysis of 5-MTP reveals positive staining in murine vascular cells [13]. Results from this study indicate that vascular 5-MTP remains detectable in *ApoE*^*−/−*^ mice fed control chow but is reduced in *ApoE*^*−/−*^ mice fed HFD. Reduction of 5-MTP may be due to HFD-induced atherosclerosis. Restoration of 5-MTP by intraperitoneal injection of 5-MTP or its analog 5-MTPE attenuates atherosclerotic growth. Importantly, 5-MTP supplement suppresses atherosclerotic calcification. These results suggest that vascular 5-MTP defends against HFD-induced atherosclerosis and calcification by protecting endothelial integrity [15, 21] and controlling inflammation [13]. Our findings indicate that 5-MTP defends against HFD-induced atherosclerotic calcification by antagonizing TLR2-mediated chondrogenesis and calcification. TLR2 is recognized as an important factor in atherosclerosis and calcification [23, 24]. It is overexpressed and activated by endogenous ligands in atherosclerotic lesions [25–27]. TLR2 exerts its effect in part by eliciting expression of pro-inflammatory cytokines such as IL-6 in a p38 MAPK-dependent manner [11, 28]. Our results indicate that 5-MTP protects against TLR2-induced calcification by down-regulating IL-6 expression. Moreover, 5-MTP inhibits Pam3-induced IL-6 production in vascular SMCs via blocking p38 MAPK signaling pathway. 5-MTP was previously reported to suppress LPS-induced IL-6 production in and release from macrophages in a p38 MAPK-dependent manner [13]. It is likely that 5-MTP exerts its anti-inflammatory actions and controls inflammation-mediated events such as HFD-induced intimal calcification by blocking p38 MAPK activation.

5-MTP controls atherosclerotic calcification by blocking TLR2-mediated vascular SMC phenotypic switch to chondrocyte phenotype. 5-MTP restores expression of contractile proteins such as α-SMA and SM22α, and suppresses chondrocyte markers such as collagen II and SOX9. It was reported that TLR2-induced SMC differentiation is governed by an upregulation of IL-6 and down-regulation of OPG in vascular SMC [10]. OPG is an extracellular decoy receptor of RANKL and plays an important role in controlling RANK-mediated osteochondrogenic differentiation and calcification at the basal state [29]. By contrast, IL-6 drives SMC migration and promotes SMC chondrogenic differentiation and calcification [10, 11, 30, 31]. Thus by upregulating IL-6 expression on one hand and suppressing OPG on the others TLR2 activation induces osteochondrogenesis and calcification. In this study, our results indicate that 5-MTP selectively blocks TLR2-induced SMC IL-6 expression without altering OPG expression, which is sufficient for controlling chondrocyte differentiation and calcification. Pro-inflammatory cytokines such as IL-6, IL-1β and TNFα are active in triggering SMC osteochongenic differentiation and calcification [32, 33]. As macrophages were reported to play an important role in osteochondrogenesis and calcification of atherosclerotic lesions through IL-6 and other cytokine productions [34], 5-MTP may exert its effects through inhibition of macrophage activation. Macrophages are major cells in the atherosclerotic plaque and play central roles in the progression of atherosclerosis. Their quantity and inflammatory phenotype in atherosclerotic plaques are major factors in controlling atherosclerosis progression. Macrophage infiltration in atherosclerotic lesions dependents on circulating monocyte/macrophages recruitment and their differentiation and proliferation [35, 36]. Thus, blocking the recruitment and differentiation of circulating monocyte/macrophages and inhibiting macrophage proliferation are potential therapeutic steps for preventing atherosclerosis progression and development of atherosclerotic calcification. As 5-MTP has been shown to reduce macrophage infiltration in lung tissues of LPS-treated mice and attenuate macrophage activation and cytokine release [13], its effect on atherosclerotic calcification may be mediated by inhibiting monocyte recruitment and macrophage activation and proliferation. Furthermore, vascular endothelial cell derived 5-MTP in circulating blood provides additional protection against monocyte recruitment and differentiation. It is therefore highly possible that 5-MTP reduces atherosclerotic calcification by controlling monocyte/macrophages recruitment, differentiation, and activation.

TLR2- and TLR4-mediated IL-6 production is mediated via p38 MAPK and CREB [11, 37]. Results from this study show that 5-MTP inhibits Pam3-induced p38 MAPK and CREB activation in vascular SMC. Furthermore, it blocks p38 MAPK and CREB activation in HFD-induced atherosclerosis in *ApoE*^*−/−*^ mice. This is in keeping with the reported data that p38 MAPK is a common signaling pathway via which 5-MTP inhibits diverse inflammatory responses in ECs, SMCs and macrophages [13, 15, 21]. The mechanism by which 5-MTP inhibits p38 MAPK remains to be elucidated.

Our results suggest that reduction of vascular 5-MTP in HFD-induced atherosclerosis is attributed to suppression of TPH-1 expression. As we did not investigate regulation of TPH-1 expression by TLR2 at the cellular and molecular level, it is unclear whether 5-MTP inhibits TPH-1 expression in vascular SMCs. It is also unclear whether TPH-1 expression is directly down-regulated by TLR2 activation or by pro-inflammatory cytokines. Previous reports suggest that LPS, IL-1β, and TNFα suppress TPH-1 expression in ECs [13, 21]. Maeda et al. reported that IL-1β and TNFα suppress TPH-1 mRNA expression in fibroblast-like synovial cells [38]. It is possible that TLR2/4 activation suppress TPH-1 expression at the transcriptional level in HFD-induced atherosclerosis. These findings suggest that vascular production of 5-MTP defends against the harmful effect of HFD. Continuous bombardment by HFD leads to exhaustion of 5-MTP and breach the defense by 5-MTP. To ameliorate the harmful effect of HFD, an obvious approach is to avoid HFD. In addition, a stable production of 5-MTP despite HFD may overcome the all effect of HFD. One experimental approach is to create 5-MTP high-producing transgenic mice by overexpression of 5-MTP synthetic enzymes. Work is in progress to test the effect of 5-MTP supermice on arterial injury and atherosclerosis.

## Conclusions

Our findings indicate that 5-MTP is a vascular arsenal against atherosclerosis and calcification by preventing TLR2–mediated SMC phenotypic switch to chondrocytes and the consequent calcification. 5-MTP exerts these effects by blocking p38 MAPK activation and inhibiting CREB and NF-κB transactivation activity. Given that 5-MTP is effective in reducing atherosclerotic progression and calcification, it may be a valuable lead compound for developing new chemoprevention approaches against atherosclerotic progression and calcification. For intellectual property protection, we evaluated the effect of a synthetic 5-MTP analog, i.e. 5-MTP methylesters, on atherosclerosis and calcification in this murine model. 5-MTPE inhibits atherosclerotic progression and calcification in a manner comparable to 5-MTP. As there is no effective treatment or prevention of atherosclerotic calcification, 5-MTP derivatives represent a new class of chemicals to prevent intimal calcification and would fulfill the unmet clinical need. Serum 5-MTP was reported to be a biomarker of human sepsis and post-myocardial infarction heart failure [13, 39]. Further studies are needed to determine if serum 5-MTP could serve as a theranostic marker for chemoprevention of atherosclerotic calcification.

## Supplementary Information

**Additional file 1: Fig. S1.** 5-MTP and 5-MTPE reduce High fat diet (HFD)-induced plaque size in ApoE − / − mice. (A) Schematic structure of the L-5MTPE. (B) paraffin-embedded aorta tissues were prepared from ApoE − / − fed chow diet or HFD with intraperitoneal injection of sline, (23.5 mg/kg), or L-5MTPE (24.7 mg/kg) twice weekly for 20 wk, and plaque size was visualized with elastin staining (200X). (C) Quantification of plaque size by ImageJ software, and quantitative results represent as mean ± SEM (n = 5 HFD-saline; n = 5 L-5MTP; n = 3 L-5MTPE). An: adventitia Lu: lumen; Med: media; Neo: neointima. (D-E) HFD-fed ApoE − / − mice were received intraperitoneal injection of saline (n = 15) or DL-5MTP (23.5 mg/kg; n = 16) twice weekly for 20 wk. (D) Vascular calcifications in aortic arch tissues were visualized with Alizarin Red S (ARS) staining and (F) quantified using ImageJ software and expressed as the percent positive area out of the total tissue area. P < 0.05, P < 0.01, P < 0.001 determined by t-test or a one-way ANOVA. **Fig. S2.** 5-MTPE suppresses collagen II expression in atherosclerotic lesions. (A) Collagen II contents in aorta tissues from ApoE − / − mice fed chow diet with saline (n = 6) or HFD with saline (n = 6) or L-5-MTPE (n = 3) twice weekly for 20 wk were analyzed by immunohistochemistry. Negative control represents staining with a 2nd antibody. (B) Immunopositive areas in the aorta tissues were quantified as a percentage of the total aortic area in each section. Data represent mean ± SEM. An: adventitia Lu: lumen; Med: media; Neo: neointima. P < 0.05, P < 0.01 determined by one-way ANOVA. **Fig. S3.** 5-MTPE decreases HFD-induced SOX9 expression in aortic atherosclerotic lesions. SOX9 and osterix contents in aorta tissues from ApoE − / − mice fed chow diet with saline (n = 3) or HFD with saline (n = 6), or L-5-MTPE (24.7 mg/kg, n = 3) twice weekly for 20 wk were analyzed by immunohistochemistry. Negative control represents staining with a 2nd antibody. Representative microphotographs (400X) of the immunohistochemical staining for (A) SOX9 and (C) osterix are shown. Immunopositive areas of (B) SOX9 and (D) osterix in aorta tissues were quantified as a percentage of the total aortic area in each section. Data represent mean ± SEM. P < 0.05 determined by a one-way ANOVA. **Fig. S4.** 5-MTPE preserve the contractile phenotype of VSMCs. (A) VSMCs were treated with different concentrations of 5-MTP for 24 h. Migration assays were then performed with PDGF-BB as a chemoattractant. (B-D) After pretreating VSMCs with 5MTP (100 μM) for 30 min, cells were stimulated with LPS for 24 h. (B) Cell lysates were immunoblotted with antibodies for α-SMA (α-smooth muscle actin), SM22 α (smooth muscle protein 22 α), or β-actin. Densitometric analysis of α-SMA and SM22 α immunoblots normalized to β-actin presented in (C) and (D). (E) After pretreating VSMCs with different concentrations of 5-MTP for 30 min, cells were stimulated with oxLDL for 24 h. Migration assays were then performed with PDGF-BB as a chemoattractant. The experiments were repeated 2 or 3 times with similar results. **Fig. S5.** 5-MTPE preserve the contractile phenotype of VSMCs. α-SMA and SM22α contents in aorta tissues from ApoE − / − mice fed chow diet with saline (n = 6) or HFD with saline (n = 6) or L-5-MTPE (n = 3) twice weekly for 20 wk were analyzed by immunohistochemistry. Negative control represents staining with a 2nd antibody. Representative microphotographs (400X) shown in (A). Immunopositive areas of (B) α-SMA and (C SM22α in the aorta tissues were quantified as a percentage of total aortic area in each section. Data in B-C represent mean ± SEM. An: adventitia Lu: lumen; Med: media; Neo: neointima. P < 0.05, P < 0.01, P < 0.001 determined by a one-way ANOVA. **Fig. S6.** 5-MTPE decreases IL-6 production in atherosclerotic lesions. Chow diet–fed or HFD-fed ApoE − / − were treated with saline or L-5-MTPE (24.7 mg/kg) twice weekly for 20 weeks. (A-B) IL-6 and (C-D) OPG in paraffin-embedded aortic tissues were analyzed by immunohistochemistry. Negative control represents staining with a 2nd antibody. Representative microphotographs (400X) of the immunohistochemical staining for (A) IL-6 and (C) OPG are shown. Immunopositive areas of (B) IL-6 and (D) OPG in aorta tissues were quantified as a percentage of total aortic area in each section. Data represent mean ± SEM (Chow + saline group: n = 6, HFD + saline group: n = 6 and HFD + 5-MTPE group: n = 3). Lu, lumen; Neo, neo-intima; Med media; An, adventitia. P < 0.05, P < 0.01 determined by a one-way ANOVA. **Fig. S7.** 5-MTP suppresses LPS-induced IL-6 production in VSMCs. After pretreating VSMCs with DMSO, DL-5-MTP or L-5-MTP (100 μM) for 30 min, VSMCs were treated with vehicle or Pam3CSK4 (Pam3) in calcifying medium for 24 h. (A) IL6 and (B) OPG level in culture supernatants was measured by ELISA. Data represent mean ± SD of at least 3 independent experiments. P < 0.01 determined by a one-way ANOVA. **Fig. S8. **5-MTP suppresses HSP60-induced VSMC migration. After pretreating VSMCs with different concentrations of 5-MTP for 30 min, cells were stimulated with HSP 60 for 24 h. Migration assays were then performed with PDGF-BB as a chemoattractant. The experiments were repeated 2 times with similar results. **Fig. S9.** 5-MTP dose-dependently suppresses Pam3-induced pp38. After pretreating VSMCs with different concentrations of L-5-MTP for 30 min, cells were stimulated with Pam3CSK4 for 30 min. Cell lysates were immunoblotted with antibodies for pp38, p38, or β-actin. The experiments were repeated 2 times with similar results.

## Data Availability

All data supporting the results reported in the article are included in this published article and its supplementary information files.

## Abbreviations

5-MTP: 5-Methoxytryptophan
5-MTPE: 5-MTP methylester
ARS: Alizarin red S
HFD: Fed high-fat diet
LPS: Lipopolysaccharide
Pam: Pam3CSK4
TLR: Toll-like receptor
TNFα: Tumor necrosis factor α
TPH1: Tryptophan hydroxylase-1
VSMC: Vascular smooth muscle cell

## References

[1] Zhu D, Mackenzie NC, Farquharson C, Macrae VE (2012). Mechanisms and clinical consequences of vascular calcification. Front Endocrinol (Lausanne).

[2] Barrett HE, Van der Heiden K, Farrell E, Gijsen FJH, Akyildiz AC (2019). Calcifications in atherosclerotic plaques and impact on plaque biomechanics. J Biomech.

[3] Jinnouchi H, Sato Y, Sakamoto A, Cornelissen A, Mori M, Kawakami R, Gadhoke NV, Kolodgie FD, Virmani R, Finn AV (2020). Calcium deposition within coronary atherosclerotic lesion: implications for plaque stability. Atherosclerosis.

[4] Shi X, Gao J, Lv Q, Cai H, Wang F, Ye R, Liu X (2020). Calcification in atherosclerotic plaque vulnerability: friend or foe?. Front Physiol.

[5] Shao JS, Cheng SL, Sadhu J, Towler DA (2010). Inflammation and the osteogenic regulation of vascular calcification: a review and perspective. Hypertension.

[6] Doherty TM, Asotra K, Fitzpatrick LA, Qiao JH, Wilkin DJ, Detrano RC, Dunstan CR, Shah PK, Rajavashisth TB (2003). Calcification in atherosclerosis: bone biology and chronic inflammation at the arterial crossroads. Proc Natl Acad Sci U S A.

[7] Sage AP, Tintut Y, Demer LL (2010). Regulatory mechanisms in vascular calcification. Nat Rev Cardiol.

[8] Durham AL, Speer MY, Scatena M, Giachelli CM, Shanahan CM (2018). Role of smooth muscle cells in vascular calcification: implications in atherosclerosis and arterial stiffness. Cardiovasc Res.

[9] Alves RD, Eijken M, van de Peppel J, van Leeuwen JP (2014). Calcifying vascular smooth muscle cells and osteoblasts: independent cell types exhibiting extracellular matrix and biomineralization-related mimicries. BMC Genomics.

[10] Lee GL, Yeh CC, Wu JY, Lin HC, Wang YF, Kuo YY, Hsieh YT, Hsu YJ, Kuo CC (2019). TLR2 promotes vascular smooth muscle cell chondrogenic differentiation and consequent calcification via the concerted actions of osteoprotegerin suppression and IL-6-mediated RANKL induction. Arterioscler Thromb Vasc Biol.

[11] Lee GL, Chang YW, Wu JY, Wu ML, Wu KK, Yet SF, Kuo CC (2012). TLR 2 induces vascular smooth muscle cell migration through cAMP response element-binding protein-mediated interleukin-6 production. Arterioscler Thromb Vasc Biol.

[12] Chava KR, Karpurapu M, Wang D, Bhanoori M, Kundumani-Sridharan V, Zhang Q, Ichiki T, Glasgow WC, Rao GN (2009). CREB-mediated IL-6 expression is required for 15(S)-hydroxyeicosatetraenoic acid-induced vascular smooth muscle cell migration. Arterioscler Thromb Vasc Biol.

[13] Wang YF, Hsu YJ, Wu HF, Lee GL, Yang YS, Wu JY, Yet SF, Wu KK, Kuo CC (2016). Endothelium-derived 5-methoxytryptophan is a circulating anti-inflammatory molecule that blocks systemic inflammation. Circ Res.

[14] Cheng HH, Kuo CC, Yan JL, Chen HL, Lin WC, Wang KH, Tsai KK, Guven H, Flaberg E, Szekely L (2012). Control of cyclooxygenase-2 expression and tumorigenesis by endogenous 5-methoxytryptophan. Proc Natl Acad Sci U S A.

[15] Ho YC, Wu ML, Su CH, Chen CH, Ho HH, Lee GL, Lin WS, Lin WY, Hsu YJ, Kuo CC (2016). A novel protective function of 5-methoxytryptophan in vascular injury. Sci Rep.

[16] Daugherty A, Tall AR, Daemen M, Falk E, Fisher EA, Garcia-Cardena G, Lusis AJ, Owens AP, Rosenfeld ME, Virmani R (2017). Recommendation on design, execution, and reporting of animal atherosclerosis studies: a scientific statement from the American Heart Association. Circ Res.

[17] Chen HL, Yuan CY, Cheng HH, Chang TC, Huang SK, Kuo CC, Wu KK (2018). Restoration of hydroxyindole O-methyltransferase levels in human cancer cells induces a tryptophan-metabolic switch and attenuates cancer progression. J Biol Chem.

[18] Wu JY, Huang TW, Hsieh YT, Wang YF, Yen CC, Lee GL, Yeh CC, Peng YJ, Kuo YY, Wen HT (2020). Cancer-derived succinate promotes macrophage polarization and cancer metastasis via succinate receptor. Mol Cell.

[19] Wu JY, Kuo CC (2012). Pivotal role of ADP-ribosylation factor 6 in toll-like receptor 9-mediated immune signaling. J Biol Chem.

[20] Wu JY, Kuo CC (2015). ADP-ribosylation factor 3 mediates cytidine-phosphate-guanosine oligodeoxynucleotide-induced responses by regulating toll-like receptor 9 trafficking. J Innate Immun.

[21] Chu LY, Wang YF, Cheng HH, Kuo CC, Wu KK (2016). Endothelium-derived 5-methoxytryptophan protects endothelial barrier function by blocking p38 MAPK activation. PLoS ONE.

[22] Da Costa CU, Wantia N, Kirschning CJ, Busch DH, Rodriguez N, Wagner H, Miethke T (2004). Heat shock protein 60 from Chlamydia pneumoniae elicits an unusual set of inflammatory responses via toll-like receptor 2 and 4 in vivo. Eur J Immunol.

[23] Edfeldt K, Swedenborg J, Hansson GK, Yan ZQ (2002). Expression of toll-like receptors in human atherosclerotic lesions: a possible pathway for plaque activation. Circulation.

[24] Chavez-Sanchez L, Garza-Reyes MG, Espinosa-Luna JE, Chavez-Rueda K, Legorreta-Haquet MV, Blanco-Favela F (2014). The role of TLR2, TLR4 and CD36 in macrophage activation and foam cell formation in response to oxLDL in humans. Hum Immunol.

[25] Roshan MH, Tambo A, Pace NP (2016). The role of TLR2, TLR4, and TLR9 in the pathogenesis of atherosclerosis. Int J Inflam.

[26] Hansson GK, Hermansson A (2011). The immune system in atherosclerosis. Nat Immunol.

[27] Lee GL, Wu JY, Yeh CC, Kuo CC (2016). TLR4 induces CREB-mediated IL-6 production via upregulation of F-spondin to promote vascular smooth muscle cell migration. Biochem Biophys Res Commun.

[28] Goulopoulou S, McCarthy CG, Webb RC (2016). Toll-like receptors in the vascular system: sensing the dangers within. Pharmacol Rev.

[29] Van Campenhout A, Golledge J (2009). Osteoprotegerin, vascular calcification and atherosclerosis. Atherosclerosis.

[30] Callegari A, Coons ML, Ricks JL, Rosenfeld ME, Scatena M (2014). Increased calcification in osteoprotegerin-deficient smooth muscle cells: dependence on receptor activator of NF-kappaB ligand and interleukin 6. J Vasc Res.

[31] Yeh CC, Wu JY, Lee GL, Wen HT, Lin P, Kuo CC (2019). Vanadium derivative exposure promotes functional alterations of VSMCs and consequent atherosclerosis via ROS/p38/NF-kappaB-mediated IL-6 production. Int J Mol Sci.

[32] Ceneri N, Zhao L, Young BD, Healy A, Coskun S, Vasavada H, Yarovinsky TO, Ike K, Pardi R, Qin L (2017). Rac2 modulates atherosclerotic calcification by regulating macrophage interleukin-1beta production. Arterioscler Thromb Vasc Biol.

[33] Al-Aly Z, Shao JS, Lai CF, Huang E, Cai J, Behrmann A, Cheng SL, Towler DA (2007). Aortic Msx2-Wnt calcification cascade is regulated by TNF-alpha-dependent signals in diabetic Ldlr-/- mice. Arterioscler Thromb Vasc Biol.

[34] Tintut Y, Patel J, Territo M, Saini T, Parhami F, Demer LL (2002). Monocyte/macrophage regulation of vascular calcification in vitro. Circulation.

[35] Moore KJ, Koplev S, Fisher EA, Tabas I, Bjorkegren JLM, Doran AC, Kovacic JC (2018). Macrophage trafficking, inflammatory resolution, and genomics in atherosclerosis: JACC macrophage in CVD series (Part 2). J Am Coll Cardiol.

[36] Barrett TJ (2020). Macrophages in atherosclerosis regression. Arterioscler Thromb Vasc Biol.

[37] Lee GL, Wu JY, Tsai CS, Lin CY, Tsai YT, Lin CS, Wang YF, Yet SF, Hsu YJ, Kuo CC (2016). TLR4-activated MAPK-IL-6 axis regulates vascular smooth muscle cell function. Int J Mol Sci.

[38] Maeda T, Miura Y, Fukuda K, Hayashi S, Kurosaka M (2015). Decoy receptor 3 regulates the expression of tryptophan hydroxylase 1 in rheumatoid synovial fibroblasts. Mol Med Rep.

[39] Lin YH, Kuo CC, Lee CM, Chou CH, Chen YH, Yeh JF, Huang CC, Hung CS, Liu LD, Ho YL (2016). 5-Methoxytryptophan is a potential marker for post-myocardial infarction heart failure—a preliminary approach to clinical utility. Int J Cardiol.

