# Supplementary material for: Restoration of 5-methoxytryptophan protects against atherosclerotic chondrogenesis and calcification in ApoE−/− mice fed high fat diet
Source: J Biomed Sci. 2021 Nov 8;28:74. doi: 10.1186/s12929-021-00771-1 (PMC8573875; doi:10.1186/s12929-021-00771-1)
Supplement: Supplementary file 1 — Additional file 1: Fig. S1. 5-MTP and 5-MTPE reduce High fat diet (HFD)-induced plaque size in ApoE − / − mice. (A) Schematic structure of the L-5MTPE. (B) paraffin-embedded aorta tissues were prepared from ApoE − / − fed chow diet or HFD with intraperitoneal injection of sline, (23.5 mg/kg), or L-5MTPE (24.7 mg/kg) twice weekly for 20 wk, and plaque size was visualized with elastin staining (200X). (C) Quantification of plaque size by ImageJ software, and quantitative results represent as mean ± SEM (n = 5 HFD-saline; n = 5 L-5MTP; n = 3 L-5MTPE). An: adventitia Lu: lumen; Med: media; Neo: neointima. (D-E) HFD-fed ApoE − / − mice were received intraperitoneal injection of saline (n = 15) or DL-5MTP (23.5 mg/kg; n = 16) twice weekly for 20 wk. (D) Vascular calcifications in aortic arch tissues were visualized with Alizarin Red S (ARS) staining and (F) quantified using ImageJ software and expressed as the percent positive area out of the total tissue area. P < 0.05, P < 0.01, P < 0.001 determined by t-test or a one-way ANOVA. Fig. S2. 5-MTPE suppresses collagen II expression in atherosclerotic lesions. (A) Collagen II contents in aorta tissues from ApoE − / − mice fed chow diet with saline (n = 6) or HFD with saline (n = 6) or L-5-MTPE (n = 3) twice weekly for 20 wk were analyzed by immunohistochemistry. Negative control represents staining with a 2nd antibody. (B) Immunopositive areas in the aorta tissues were quantified as a percentage of the total aortic area in each section. Data represent mean ± SEM. An: adventitia Lu: lumen; Med: media; Neo: neointima. P < 0.05, P < 0.01 determined by one-way ANOVA. Fig. S3. 5-MTPE decreases HFD-induced SOX9 expression in aortic atherosclerotic lesions. SOX9 and osterix contents in aorta tissues from ApoE − / − mice fed chow diet with saline (n = 3) or HFD with saline (n = 6), or L-5-MTPE (24.7 mg/kg, n = 3) twice weekly for 20 wk were analyzed by immunohistochemistry. Negative control represents staining with a 2nd antibody. R [file 12929_2021_771_MOESM1_ESM.pdf]

Supplementary Figure 1

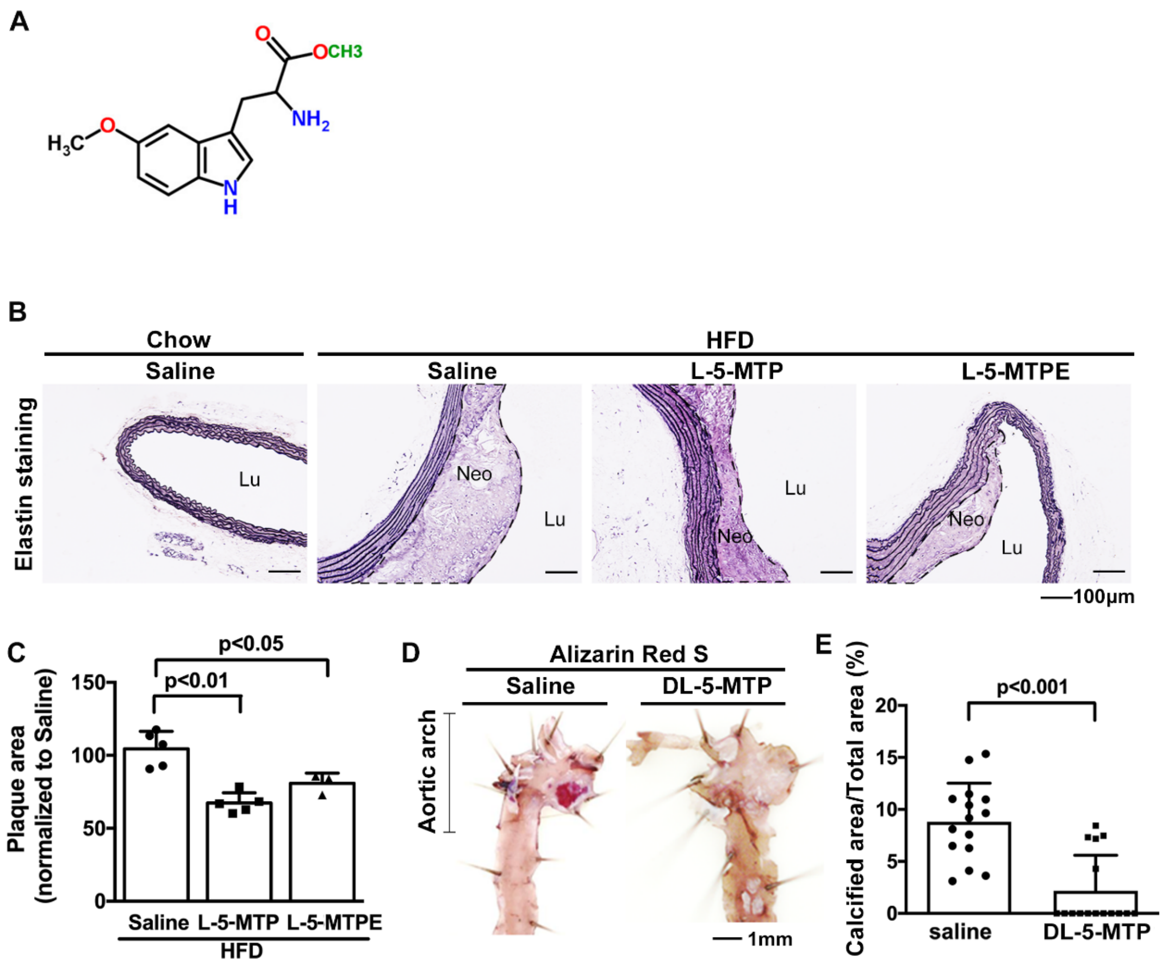

Supplementary Fig. 1. 5-MTP and 5-MTPE reduce High fat diet (HFD)-induced plaque size in *ApoE*$^{-/-}$ mice. (A) Schematic structure of the L-5MTPE. (B) paraffin-embedded aorta tissues were prepared from *ApoE*$^{-/-}$ fed chow diet or HFD with intraperitoneal injection of sline, (23.5 mg/kg), or L-5MTPE (24.7 mg/kg) twice weekly for 20 wk, and plaque size was visualized with elastin staining (200X). (C) Quantification of plaque size by ImageJ software, and quantitative results represent as mean ± SEM (n = 5 HFD-saline; n = 5 L-5MTP; n = 3 L-5MTPE). An: adventitia Lu: lumen; Med: media; Neo: neointima. (D-E) HFD-fed *ApoE*$^{-/-}$ mice were received intraperitoneal injection of saline (n=15) or DL-5MTP (23.5 mg/kg; n=16) twice weekly for 20 wk. (D) Vascular calcifications in aortic arch tissues were visualized with Alizarin Red S (ARS) staining and (F) quantified using ImageJ software and expressed as the percent positive area out of the total tissue area. $P < 0.05$, $P < 0.01$, $P < 0.001$ determined by *t*-test or a one-way ANOVA.

Supplementary Figure 2

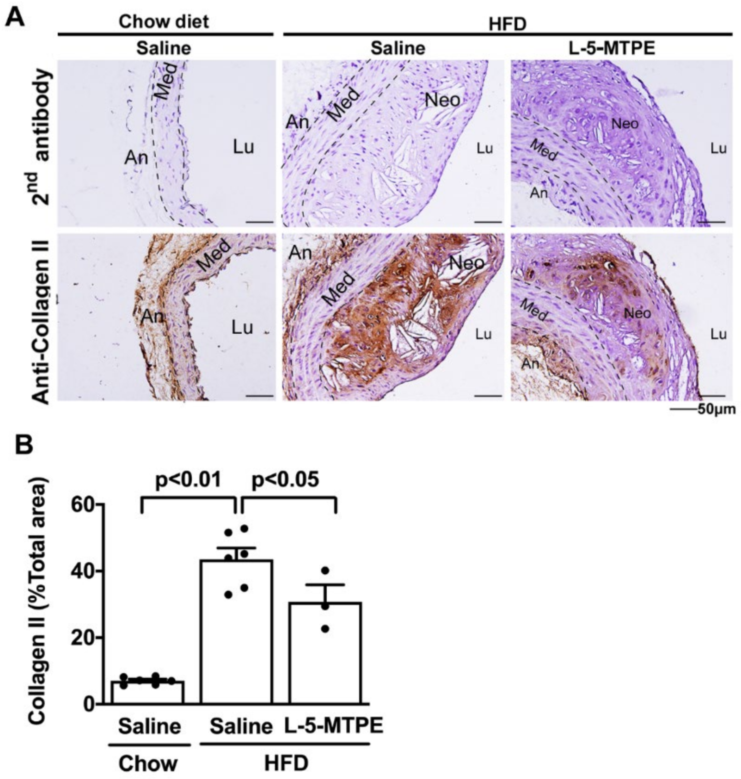

Supplementary Fig. 2. 5-MTPE suppresses collagen II expression in atherosclerotic lesions. (A) Collagen II contents in aorta tissues from *ApoE*$^{-/-}$mice fed chow diet with saline (n= 6) or HFD with saline (n = 6) or L-5-MTPE (n = 3) twice weekly for 20 wk were analyzed by immunohistochemistry. Negative control represents staining with a 2nd antibody. (B) Immunopositive areas in the aorta tissues were quantified as a percentage of the total aortic area in each section. Data represent mean ± SEM. An: adventitia Lu: lumen; Med: media; Neo: neointima. $P < 0.05$, $P < 0.01$ determined by one-way ANOVA.

Supplementary Figure 3

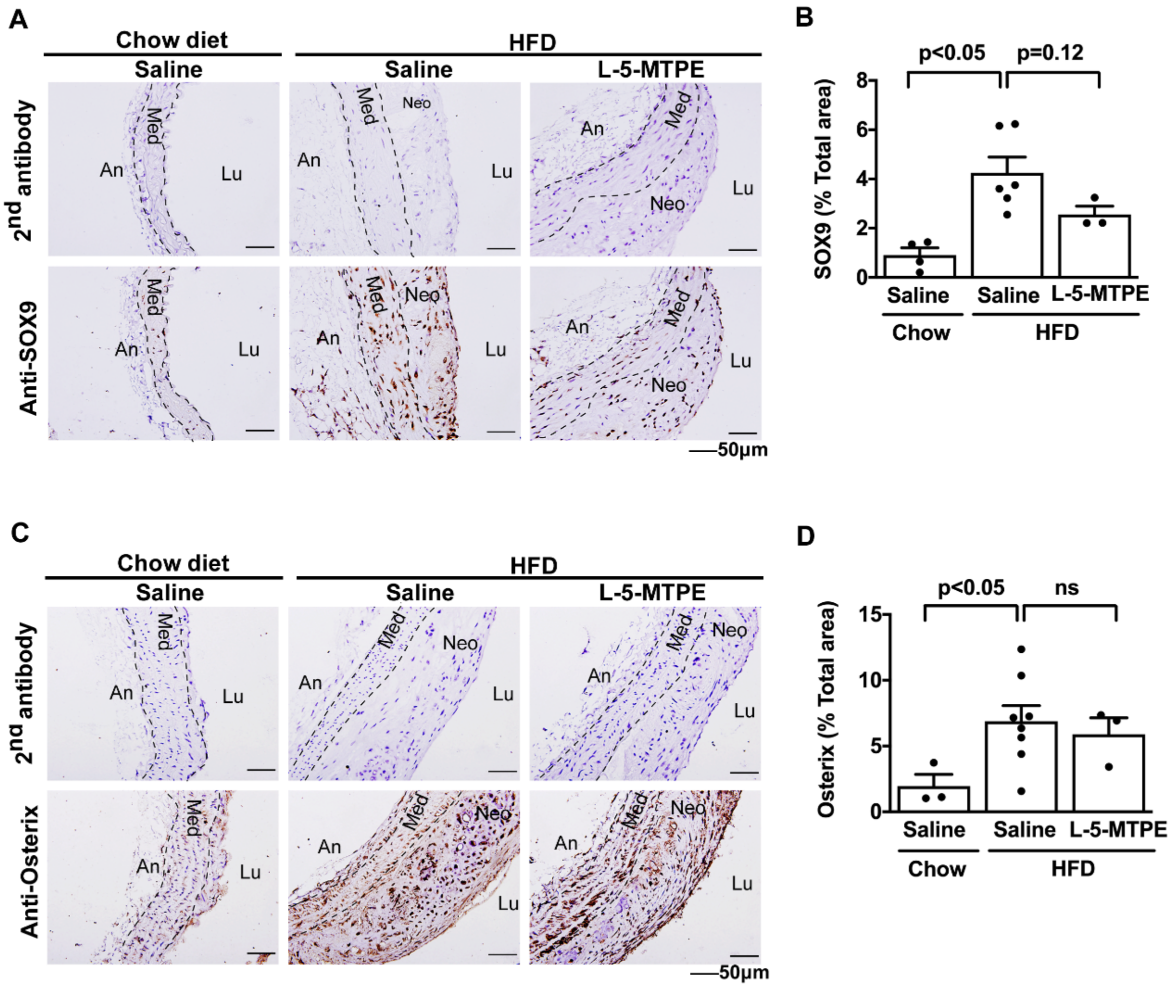

Supplementary Fig. 3. 5-MTPE decreases HFD-induced SOX9 expression in aortic atherosclerotic lesions. SOX9 and osterix contents in aorta tissues from *ApoE*$^{-/-}$mice fed chow diet with saline (n= 3) or HFD with saline (n = 6), or L-5-MTPE (24.7 mg/kg, n = 3) twice weekly for 20 wk were analyzed by immunohistochemistry. Negative control represents staining with a 2nd antibody. Representative microphotographs (400X) of the immunohistochemical staining for (A) SOX9 and (C) osterix are shown. Immunopositive areas of (B) SOX9 and (D) osterix in aorta tissues were quantified as a percentage of the total aortic area in each section. Data represent mean ± SEM. $P < 0.05$ determined by a one-way ANOVA.

Supplementary Figure 4

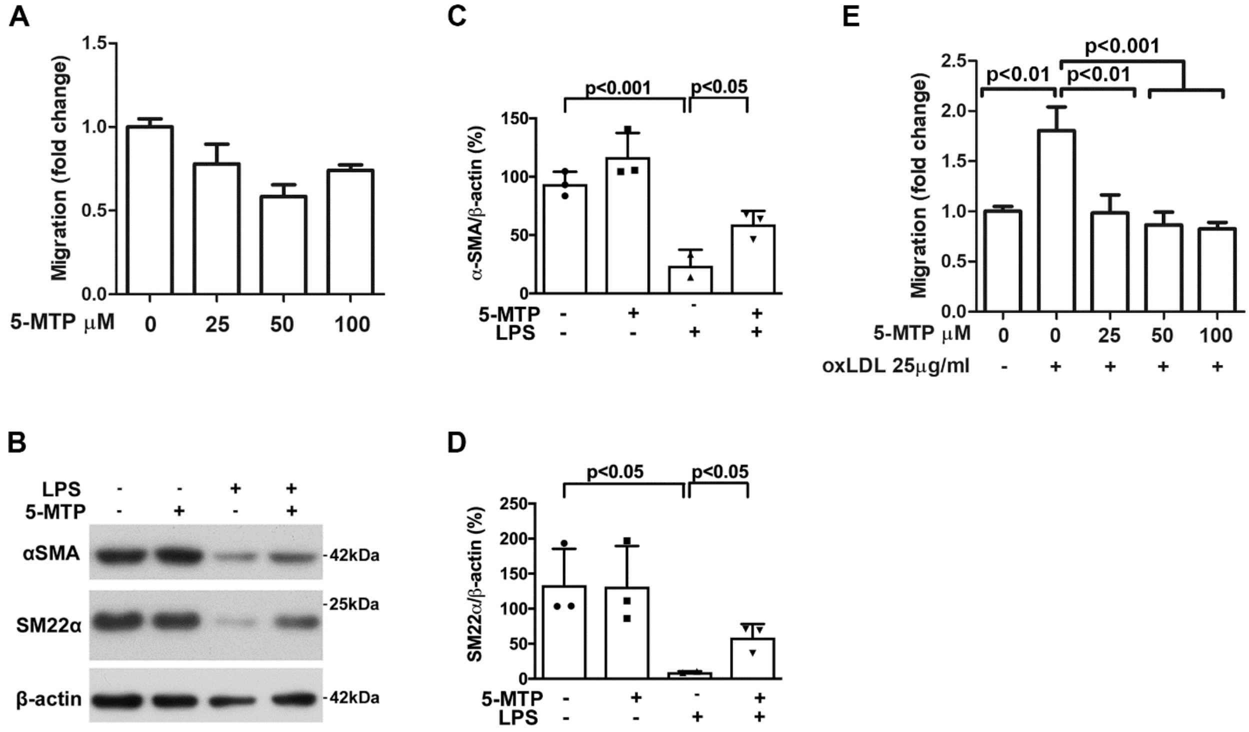

Supplementary Fig. 4. 5-MTPE preserve the contractile phenotype of VSMCs. (A) VSMCs were treated with different concentrations of 5-MTP for 24 h. Migration assays were then performed with PDGF-BB as a chemoattractant. (B-D) After pretreating VSMCs with 5MTP (100 μM) for 30 minutes, cells were stimulated with LPS for 24 hours. (B) Cell lysates were immunoblotted with antibodies for α-SMA (α-smooth muscle actin), SM22 α (smooth muscle protein 22 α), or β -actin. Densitometric analysis of α-SMA and SM22 α immunoblots normalized to β-actin presented in (C) and (D). (E) After pretreating VSMCs with different concentrations of 5-MTP for 30 min, cells were stimulated with oxLDL for 24 h. Migration assays were then performed with PDGF-BB as a chemoattractant. The experiments were repeated 2 or 3 times with similar results.

Supplementary Figure 5

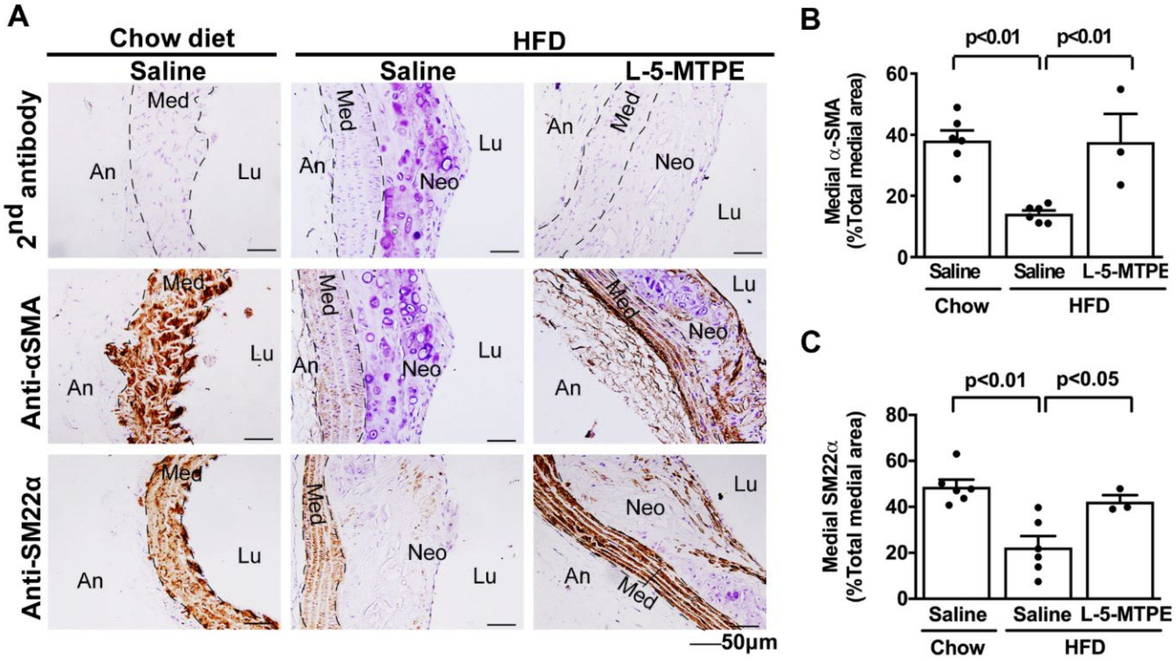

Supplementary Fig. 5. 5-MTPE preserve the contractile phenotype of VSMCs. α-SMA and SM22α contents in aorta tissues from *ApoE*$^{-/-}$ mice fed chow diet with saline (n= 6) or HFD with saline (n = 6) or L-5-MTPE (n = 3) twice weekly for 20 wk were analyzed by immunohistochemistry. Negative control represents staining with a 2nd antibody. Representative microphotographs (400X) shown in (A). Immunopositive areas of (B) α-SMA and (C SM22α in the aorta tissues were quantified as a percentage of total aortic area in each section. Data in B-C represent mean ± SEM**.** An: adventitia Lu: lumen; Med: media; Neo: neointima. $P < 0.05$, $P < 0.01$, $P < 0.001$ determined by a one-way ANOVA.

Supplementary Figure 6

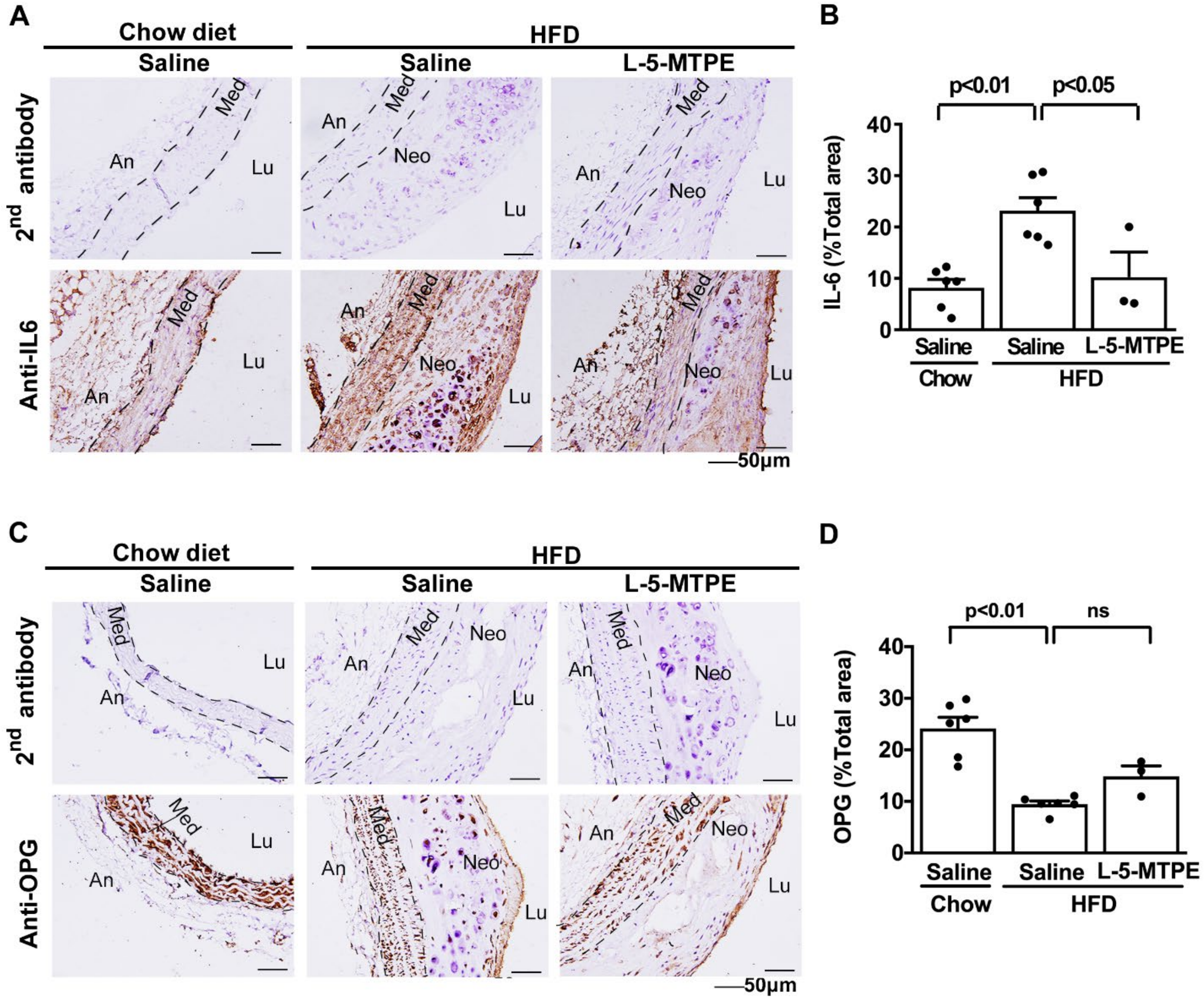

Supplementary Fig. 6. 5-MTPE decreases IL-6 production in atherosclerotic lesions. Chow diet–fed or HFD-fed *ApoE*$^{-/-}$ were treated with saline or L-5-MTPE (24.7 mg/kg) twice weekly for 20 weeks. (A-B) IL-6 and (C-D) OPG in paraffin-embedded aortic tissues were analyzed by immunohistochemistry. Negative control represents staining with a 2nd antibody. Representative microphotographs (400X) of the immunohistochemical staining for (A) IL-6 and (C) OPG are shown. Immunopositive areas of (B) IL-6 and (D) OPG in aorta tissues were quantified as a percentage of total aortic area in each section. Data represent mean ± SEM (Chow + saline group: n = 6, HFD + saline group: n = 6 and HFD + 5-MTPE group: n = 3). Lu, lumen; Neo, neo-intima; Med media; An, adventitia. $P < 0.05$, $P < 0.01$ determined by a one-way ANOVA.

Supplementary Figure 7

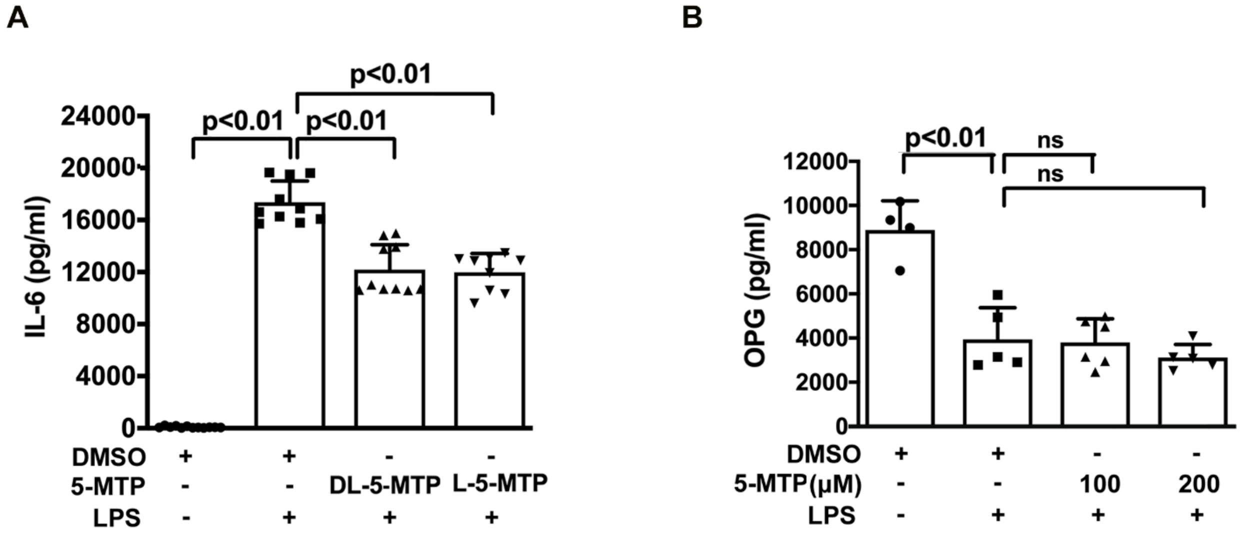

Supplementary Fig. 7. 5-MTP suppresses LPS-induced IL-6 production in VSMCs. After pretreating VSMCs with DMSO, DL-5-MTP or L-5-MTP (100 μM) for 30 minutes, VSMCs were treated with vehicle or Pam3CSK4 (Pam3) in calcifying medium for 24 h. (A) IL6 and (B) OPG level in culture supernatants was measured by ELISA. Data represent mean ± SD of at least 3 independent experiments. $P < 0.01$ determined by a one-way ANOVA.

Supplementary Figure 8

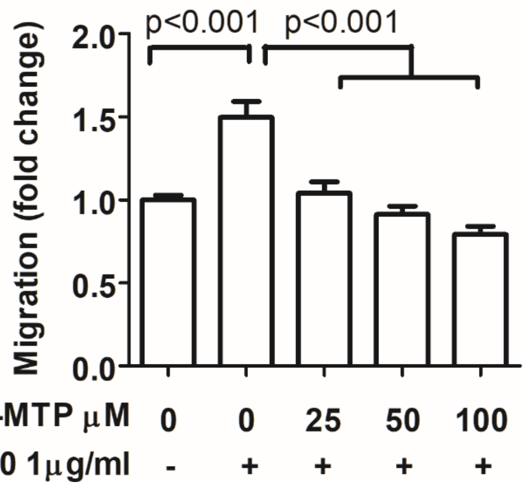

Supplementary Fig. 8. 5-MTP suppresses HSP60-induced VSMC migration. After pretreating VSMCs with different concentrations of 5-MTP for 30 min, cells were stimulated with HSP 60 for 24 h. Migration assays were then performed with PDGF-BB as a chemoattractant. The experiments were repeated 2 times with similar results.

Supplementary Figure 9

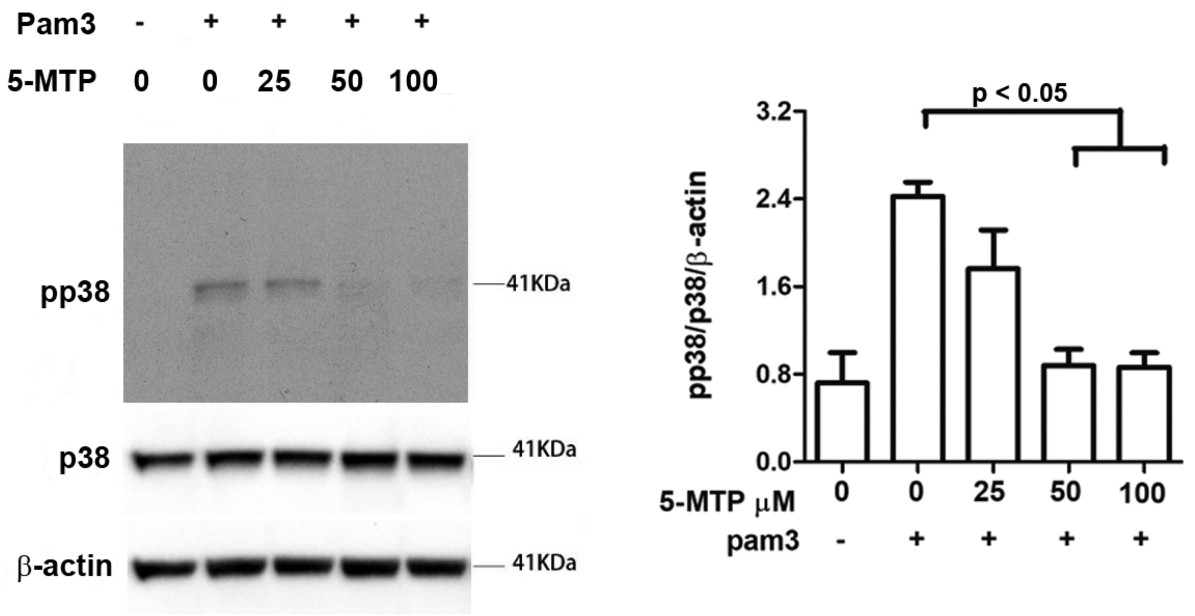

Supplementary Fig. 9. 5-MTP dose-dependently suppresses Pam3-induced pp38. After pretreating VSMCs with different concentrations of L-5-MTP for 30 min, cells were stimulated with Pam3CSK4 for 30 min. Cell lysates were immunoblotted with antibodies for pp38, p38, or β-actin. The experiments were repeated 2 times with similar results.
